# Anti-Melanoma Effects of Miconazole: Investigating the Mitochondria Involvement

**DOI:** 10.3390/ijms25073589

**Published:** 2024-03-22

**Authors:** Francesca Scatozza, Maria Miriam Giardina, Carola Valente, Virginia Vigiano Benedetti, Antonio Facchiano

**Affiliations:** Istituto Dermopatico dell’Immacolata, IDI-IRCCS, 00167 Rome, Italy

**Keywords:** mitochondria, ATP, ROS, melanoma, miconazole, clotrimazole, carnitine

## Abstract

Miconazole is an antimycotic drug showing anti-cancer effects in several cancers. However, little is known on its effects in melanoma. A375 and SK-MEL-28 human melanoma cell lines were exposed to miconazole and clotrimazole (up to 100 mM). Proliferation, viability with MTT assay and vascular mimicry were assayed at 24 h treatment. Molecular effects were measured at 6 h, namely, ATP-, ROS-release and mitochondria-related cytofluorescence. A metabolomic profile was also investigated at 6 h treatment. Carnitine was one of the most affected metabolites; therefore, the expression of 29 genes involved in carnitine metabolism was investigated in the public platform GEPIA2 on 461 melanoma patients and 558 controls. After 24 h treatments, miconazole and clotrimazole strongly and significantly inhibited proliferation in the presence of 10% serum on either melanoma cell lines; they also strongly reduced viability and vascular mimicry. After 6 h treatment, ATP reduction and ROS increase were observed, as well as a significant reduction in mitochondria-related fluorescence. Further, in A375, miconazole strongly and significantly altered expression of several metabolites including carnitines, phosphatidyl-cholines, all amino acids and several other small molecules, mostly metabolized in mitochondria. The expression of 12 genes involved in carnitine metabolism was found significantly modified in melanoma patients, 6 showing a significant impact on patients’ survival. Finally, miconazole antiproliferation activity on A375 was found completely abrogated in the presence of carnitine, supporting a specific role of carnitine in melanoma protection toward miconazole effect, and was significantly reversed in the presence of caspases inhibitors such as ZVAD-FMK and Ac-DEVD-CHO, and a clear pro-apoptotic effect was observed in miconazole-treated cells, by FACS analysis of Annexin V-FITC stained cells. Miconazole strongly affects proliferation and other biological features in two human melanoma cell lines, as well as mitochondria-related functions such as ATP- and ROS-release, and the expression of several metabolites is largely dependent on mitochondria function. Miconazole, likely acting via carnitine and mitochondria-dependent apoptosis, is therefore suggested as a candidate for further investigations in melanoma treatments.

## 1. Introduction

Melanoma is the most aggressive skin cancer. According to the American Cancer Society, about one hundred thousand new melanoma cases are diagnosed in USA each year with about eight thousand deaths, mostly in men (https://www.cancer.org/cancer/types/melanoma-skin-cancer/about/key-statistics.html, accessed on 10 March 2024). These numbers are similar in Europe. Relevant improvements have emerged recently in melanoma treatment, based on the re-activation of the immune response against the tumor (immunotherapy) and on the use of drugs targeting specific molecules (targeted therapy). Such new treatments have significantly improved overall survival; however, advanced stages of this cancer still have poor prognosis. Therefore, the identification of novel effective approaches in melanoma is still urgently needed; this is related to the high costs of the available treatments and to the recurrence risk associated to molecular adaptation mechanisms [1]. To this aim, drug repositioning strategies may represent a particularly interesting approach. Investigating, in a specific setup, drugs developed for other clinical conditions, represents a promising field, since pharmacokinetic and toxicological profiles of such drugs are known, making faster and less expensive their application in clinics, with indubitable benefits for patients and for the national health systems [2]. Miconazole has strong antimycotic activity and is known from the early seventies [3,4]. However, while clinical applications are mainly antimycotic, the mechanism of action involves inhibition of CYP450 [5,6], which plays a key role in several fundamental processes such as estrogen oxidation [7], control of metabolism and activity of many drugs [8], vitamin D metabolism [9], cell survival and proliferation [10]. A potential role of CYP450 has been suggested in several pathological conditions [11,12,13,14,15] and several cancer types [16,17,18,19,20,21,22,23,24,25,26], including melanoma [27]. This indicates how miconazole may affect several overlapping targets relevant in several pathological conditions, in addition to mycotic infections. We have recently shown for the first time that miconazole has a strong anti-melanoma effect in vitro, showing both a relevant dose-dependent inhibition of serum-induced proliferation and a marked cell death inducing effect [28]. In that study, we also showed that miconazole strongly increases its antimelanoma effect in *KCNN2* silenced cells, indicating its activity as strongly regulated by the K+ ion channel KCNN2. This is consistent with the known ability of miconazole to target ion channels [29,30]. The known property of miconazole to preferentially target the skin [31], along with studies performed to increase its skin delivery [32,33], makes this molecule a relevant drug in skin diseases and an interesting new candidate in melanoma treatment. In the present study, we investigated how human melanoma cells (namely, A375 and SK-MEL-28) respond to miconazole and to an additional antimycotic molecule, namely clotrimazole. They belong to the same Azoles antimycotic drugs; however, while they may share common targets, strong differences in the biological effects are expected due to the different number of chlorides and rings and the presence of the oxygen at the hinge of the rings. We investigated biological response after 24 h treatment, namely proliferation, viability, and vascular mimicry. We then investigated molecular mechanisms underlying these biological effects by addressing, after 6 h treatment, mitochondria fluorescence, mitochondria-related functions such as ATP and ROS release, and the metabolic response, via a large metabolomics profile.

## 2. Results

We preliminarily investigated the antiproliferative effect of 4 azoles antifungal molecules, namely, fluconazole, voriconazole, miconazole and clotrimazole. Preliminary analyses indicated that fluconazole and voriconazole show no antiproliferative effects on either A375 and SK-MEL-28 human melanoma cells in 24 h treatment experiments at a dose range up to 100 μM (see Supplementary Materials Figure S1). On the contrary, miconazole and clotrimazole showed significant antiproliferative effects and were then investigated in further detail. Miconazole and clotrimazole effects were then investigated in more detail on human melanoma cells (A375 and SK-MEL-28), after 24 h treatments, to measure biological effects such as viability, proliferation and vascular mimicry. In addition, early effects were also investigated after 6 h treatment, to measure upstream effects likely related to the molecular mechanism of action.

### 2.1. Effects at 24 h: Viability, Proliferation, Vascular Mimicry

#### 2.1.1. Viability and Proliferation

**Figure 1:**
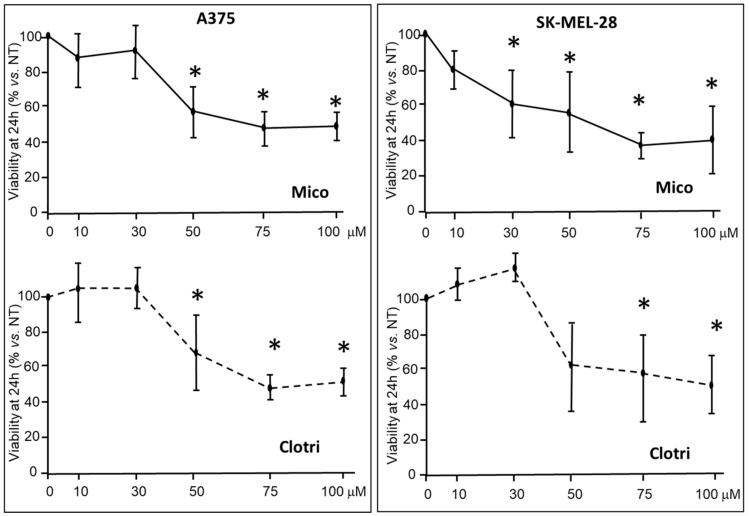
Effects of miconazole (MICO) and clotrimazole (Clotri) on cells viability by MTT after 24 h exposure, in A375 and SK-MEL-28 cells, in the presence of 10% FBS. Data are expressed as mean ± S.D. of at least three independent experiments One-way ANOVA analysis shows a significant effect in all four panels; asterisks indicate significant difference vs. 0 μM according to multiple comparisons with Dunnet multiple comparison test. (* = *p* < 0.05). The 0 μM dose corresponds to DMSO only.

A375 and SK-MEL-28 cells were treated with increasing miconazole and clotrimazole doses, in the presence of 10% FBS, and the viability was measured after 24 h treatment. As reported in Figure 1, the MTT assay, carried out as reported [34], reveals significant viability reduction in either cell types, with miconazole showing an effect similar to clotrimazole. Based on this experiment, miconazole and clotrimazole show IC50 of 32.5 and 47.9 μM, respectively.

**Figure 2:**
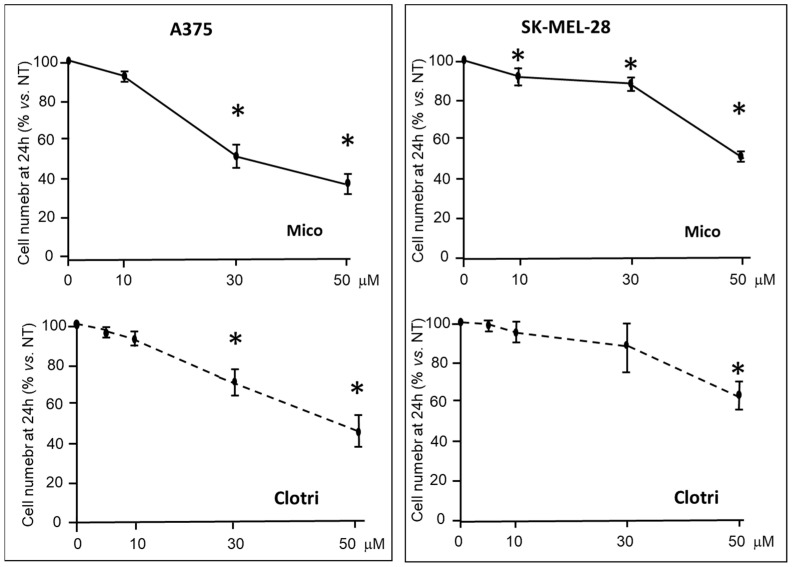
Proliferation of A375 and SK-MEL-28 melanoma cells under miconazole or clotrimazole treatment for 24 h in the presence of 10% FBS. Data are expressed as mean ± S.D. of at least three independent experiments. One-way ANOVA shows a significant effect in all four panels; asterisks indicate significant difference vs. 0 μM according to multiple comparisons with Dunnet test. (* = *p* < 0.05). The 0 μM dose corresponds to DMSO only.

In proliferation assays, A375 and SK-MEL-28 cells were treated with increasing doses of miconazole or clotrimazole in the presence of 10% FBS. Cell number was counted at 24 h. Figure 2 shows that both miconazole and clotrimazole significantly reduce cell number in a dose-dependent manner.

Supplementary Materials Figure S2 shows representative fields of A375 and SK-MEL-28 cells, respectively, at 24 h and 48 h treatments, exposed to miconazole, in dose-dependence experiments. These images show time- and dose-dependent antiproliferation effects in either cell types.

#### 2.1.2. Vasculogenic Mimicry

**Figure 3:**
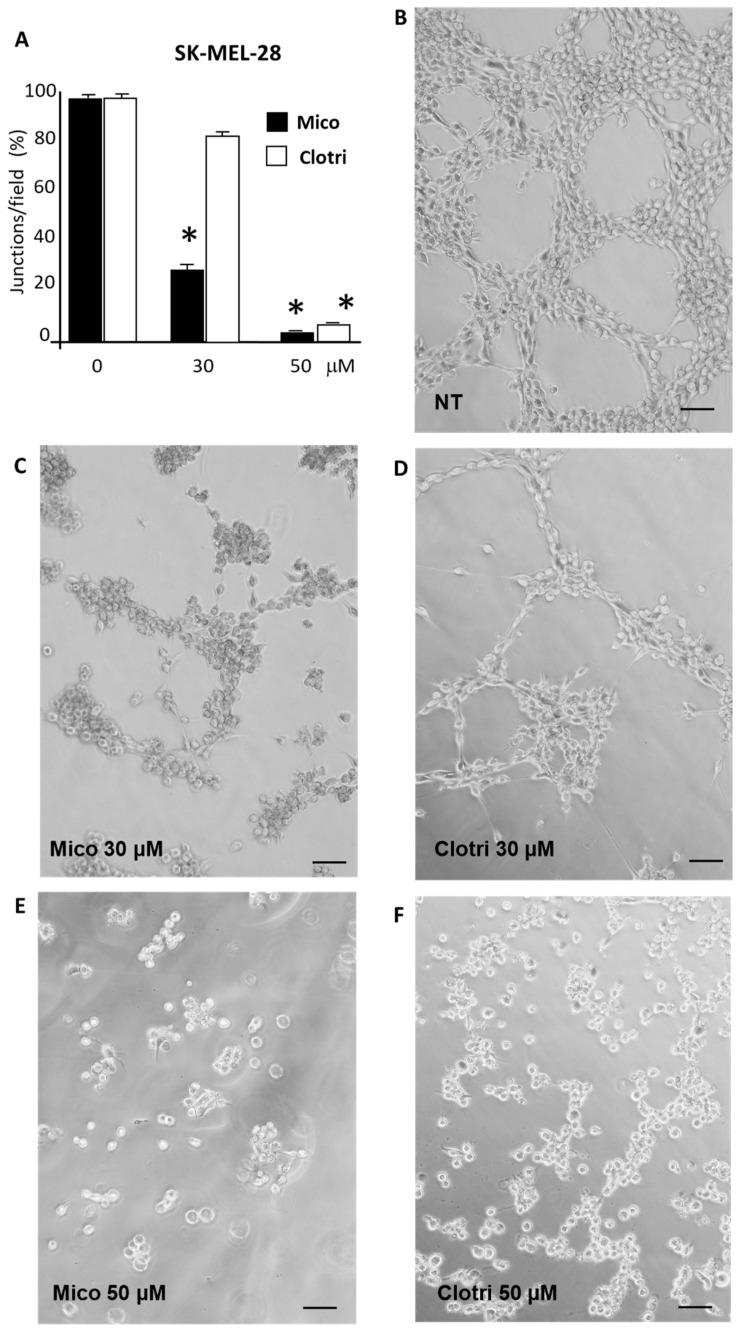
Vasculogenic mimicry of SK-MEL-28 under treatment with either miconazole or clotrimazole for 24 h. Quantification from three experiments (**A**); representative fields for not-treated (NT) (**B**), treated with miconazole 30 μM (**C**) and 50 μM (**E**), with clotrimazole 30 μM (**D**) and 50 μM (**F**) in the presence of 10% FBS. Representative images from one of at least three independent experiments are reported. Vascular mimicry (VM) in SK-MEL-28 was analyzed by counting vessel-like formations expressed as a number of closed polygons in random microscope fields. One-way ANOVA shows a significant effect for miconazole at 30 and 50 μM, and for clotrimazole at 50 μM treatment. The 0 μM dose corresponds to DMSO only. Asterisks indicate a significant effect vs. 0 μM according to multiple comparisons with Dunnet multiple comparison test. (* = *p* < 0.05). (100× magnification). Scale bar = 50 µM.

Vasculogenic mimicry is often investigated in melanoma in vitro experimental set-up, as well as in other cancer types. We then investigated the effect of miconazole and clotrimazole 24 h treatment on vessel-like structures formation. Under our experimental conditions, A375 cells show a poor vasculogenic mimicry behavior; we therefore focused on SK-MEL-28. As shown in Figure 3, under normal conditions, Matrigel-seeded cells adopt a chord-like phenotype and form an arborizing pattern, while under miconazole or clotrimazole treatment, an evident reduction in vessel-like channel formations occurs. Figure 3A reports a quantification of the arborizing pattern as indicated in the Materials and Methods section and indicates a strong and significant dose-dependent reduction in vessel-like formations under either miconazole or clotrimazole treatment. Under these experimental conditions, miconazole appears to be more potent as compared to clotrimazole.

### 2.2. Effects at 6 h: ATP, ROS, Mitochondria and Metabolomics

#### 2.2.1. ATP and ROS Levels

**Figure 4:**
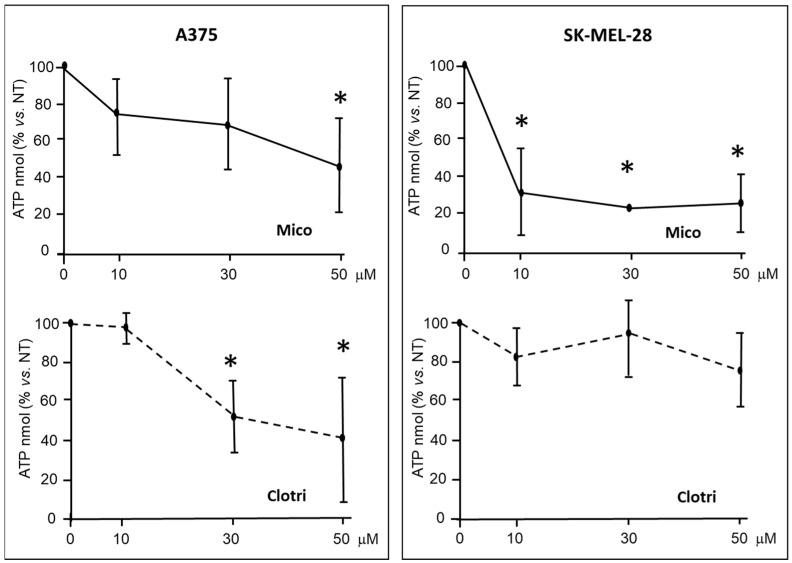
ATP levels in lysates from cells under miconazole and clotrimazole treatment for 6 h. Miconazole and clotrimazole treatment strongly reduced ATP levels in A375 and SK-MEL-28 cells after 6 h treatment. Data are expressed as mean ± S.D. of at least three independent experiments. The 0 μM dose corresponds to DMSO only. One-way ANOVA shows a significant effect in three out of four panels; asterisks indicate significant difference vs. 0 μM, according to multiple comparisons with Dunnet test. (* = *p* < 0.05).

ATP levels were measured in A375 and SK-MEL-28 cells. Figure 4 shows that ATP levels are significantly reduced upon 6 h treatment either with miconazole and clotrimazole, in a dose-dependent way. Miconazole appears as effective or more potent than clotrimazole.

**Figure 5:**
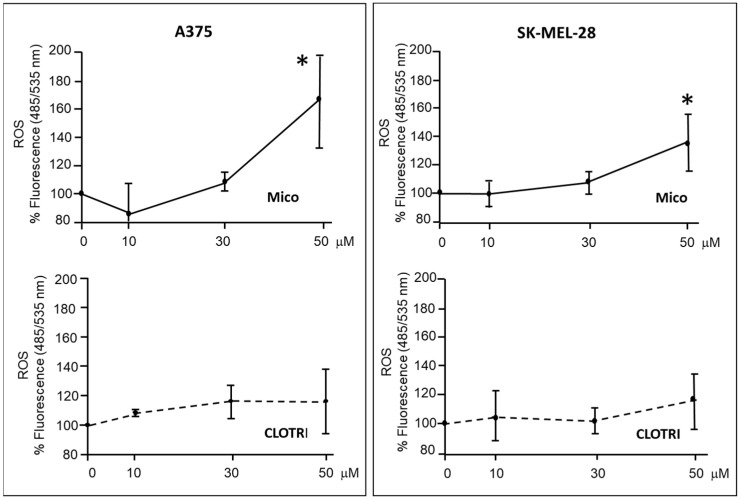
ROS levels in lysates from cells under miconazole and clotrimazole treatment for 6 h. Miconazole treatment induces a significant increase in ROS production. Data are expressed as mean ± S.D. of at least three independent experiments. The 0 μM dose corresponds to DMSO only. One-way ANOVA shows a significant effect in miconazole-treated cells; asterisks indicate significant difference vs. 0 μM, according to multiple comparisons with Dunnet test. (* = *p* < 0.05).

ROS levels were then measured on either cell types treated with miconazole and clotrimazole. Figure 5 shows dose-dependent experiments indicating a significant increase in ROS levels in A375 and SK-MEL-28 cells upon 6 h treatment with miconazole 50 μM, while clotrimazole shows a moderate not-significant effect.

#### 2.2.2. Mitochondria Investigation

**Figure 6:**
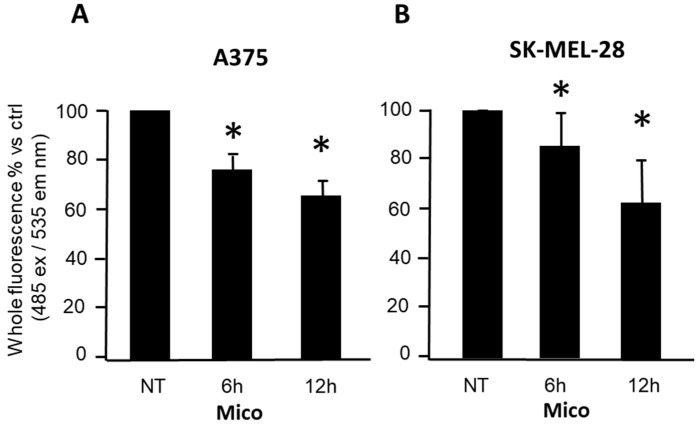
Whole fluorescence of cells stained with Biotracker™ 488 Green Mitochondria Dye. Either A375 (**A**) and SK-MEL-28 cells (**B**) were treated with miconazole 50 μM at increasing times. Miconazole treatment at 6 h and 24 h showed a significant effect in either cases as compared to ctrl (NT = DMSO-treated) according to the *t*-test (asterisks indicate *p* < 0.05).

As shown in Figure 4 and Figure 5, respectively, ATP and ROS levels are significantly modified after 6 h treatment with miconazole. This result may suggest a possible involvement of mitochondria, as discussed in the Discussion section. In preliminary experiments, SK-MEL-28 and A375 cells were then treated at different times, with miconazole and stained with the Biotracker™ 488 Green Mitochondria Dye. The whole fluorescence upon 6 h and 12 h treatment was measured at the Ensight fluorometer [35] and was found significantly reduced at 50 μM dose in either A375 cells and SK-MEL-28, as shown in Figure 6A and 6B, respectively, suggesting that miconazole may be able to significantly alter mitochondria-related cellular fluorescence as early as at 6 h treatment.

**Figure 7:**
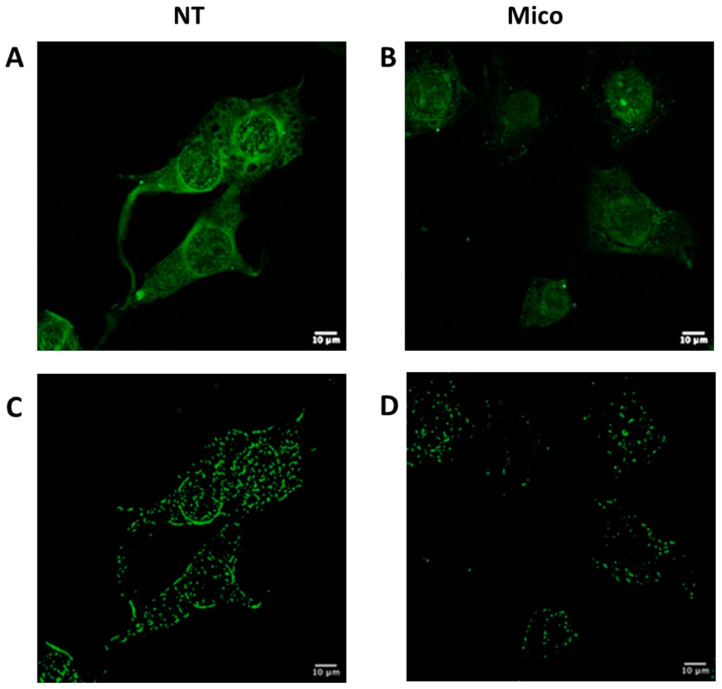
(**A**,**B**): SK-MEL-28 LSM images at 63× magnification, untreated (NT = DMSO-treated) (**A**) and treated (**B**) with miconazole 50 μM for 6 h (representative images of 30 fields). Mitochondria were stained with BioTracker 488 Green Mitochondria dye (Merck Millipore Bio, Temecula, CA, USA). (**C**,**D**): mitochondria identified using the “Mitochondria analyzer” tool.

We then investigated confocal cytofluorescence of treated and untreated cells to have a single-cell picture of intracellular fluorescence using Biotracker™ 488 Green Mitochondria Dye. Confocal cytofluorescence analysis was carried out on SK-MEL-28 cells treated with DMSO alone for 6 h as control or with miconazole 50 μM dissolved in DMSO. Figure 7 shows one representative image of untreated cells (panel A) and one representative image of miconazole-treated cells (Panel B). A change in mitochondria-related fluorescence is evident in miconazole-treated cells, at 6 h, indicating a strong reduction in mitochondria-associated fluorescence, similar to the reduction in the whole fluorescence reported in Figure 6. This effect led us to hypothesize that the reduced cytoplasmic staining is related to the loss of mitochondria integrity or reduced mitochondria number or to mitochondria size-reduction.

Panel C and D of Figure 7 report the A and B images analyzed with the “Mitochondrial analyzer” tool to identify mitochondria. This tool was used as reported in more detail in the Material and Methods section. According to this analysis, a clear reduction in mitochondria signals appears.

**Figure 8:**
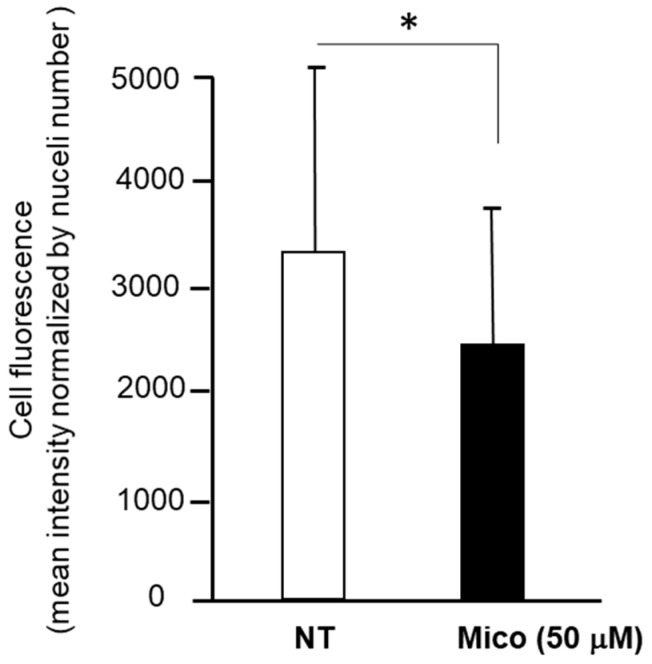
Fluorescence intensity quantification of SK-MEL-28 stained with BioTracker 488 Green Mitochondria dye (Merck Millipore Bio, Temecula, CA, USA) measured by Image J2 software on 30 randomly chosen fields from LSM images. NT = DMSO-treated. Mean + St. Dev. is reported. Asterisk indicates significant *p* value according to Student *t*-test (*p* = 0.025).

Figure 8 reports the quantification of the cytofluorescence of the experiment depicted in Figure 7 from 30 fields of miconazole-treated and 30 fields of untreated cells. It indicates a significant reduction in mitochondria-associated fluorescence by confocal cytofluorescence. Data generating this graph are reported in Table S3.

#### 2.2.3. Metabolomics Analysis

Miconazole effect on A375 melanoma cells metabolism was then investigated by measuring expression levels of metabolites and small molecules. The 30 μM dose was chosen, at 6 h treatment, vs. control (i.e., DMSO-treated cells). One thousand nineteen metabolites were under investigation; 443 were found at a level falling within the detection range (the complete list is reported in Table S1). Out of these, 83 significantly different levels of metabolites and metabolism indicators were identified, mostly carnitines, cholines, different lipid types and most amino acids. Table 1 reports all 83 significant differences identified. The multiple testing correction was performed according to Benjamini procedure [36], as detailed in the Methods section.

Within the top 15 metabolites and metabolism indicators most altered, carnitines C0, C2, C3, C4 and C5 (respectively, Carnitine, Acetylcarnitine, Propionylcarnitine, Butyrylcarnitine and Valerylcarnitine) were found significantly altered. At line 1 of Table 1, the ratio C4/C3, indicating Short-Chain Acyl-Coenzyme A Dehydrogenase Deficiency is shown as the most modified indicator (12.4-fold increase in treated samples vs. ctrl), while the third most modified (line 3) was the ratio C4/C2, indicating Isobutyryl Coenzyme A Dehydrogenase Deficiency (5.37-fold increase in treated samples vs. ctrl). In most cases, carnitine levels are much lower in the miconazole-treated cells, indicating reduced fatty acid oxidation, also indicated by the lower beta-oxidation ratio, for instance, line 38 indicates C2/C0 ratio reduced by 0.57-fold decrease in treated vs. ctrl. Other significant changes of carnitines ratio values are present (lines 51,53, 55, 78, 79, 81 in Table 1) also as absolute values of Carnitines expression (see lines 78, 81). Further, strong alteration in polyammines, putrescine and asparagine is found in the top 15 most altered metabolites, indicated in Table 1. Also, LDH activity was found largely modified (line 2 of Table 1), computed by the ratio of lactate to hexose, mainly caused by a significant reduction in hexose. This could indicate increased anaerobic glycolysis to counteract the reduced lipid oxidation. This also explains the increased glutaminolysis rate. Overall, Table 1 shows a strongly altered expression level of all amino acids, along with significant alteration of many fatty acids and poly-ammines levels. Notably, amino acids and fatty acids metabolism is mostly occurring at the mitochondrial levels, confirming an early strong alteration of mitochondria activity.

In conclusion, miconazole 6 h treatment strongly modifies the expression of many metabolites, including carnitines, many fatty acids and nearly all amino acids, in melanoma cells. Table S2 reports the metabolic role (according to documentation accompanying Biocrates MxP^®^ Quant 500 XL kit) of the indicators shown in Table 1.

#### 2.2.4. Investigating Carnitine’s Role

**Figure 9:**
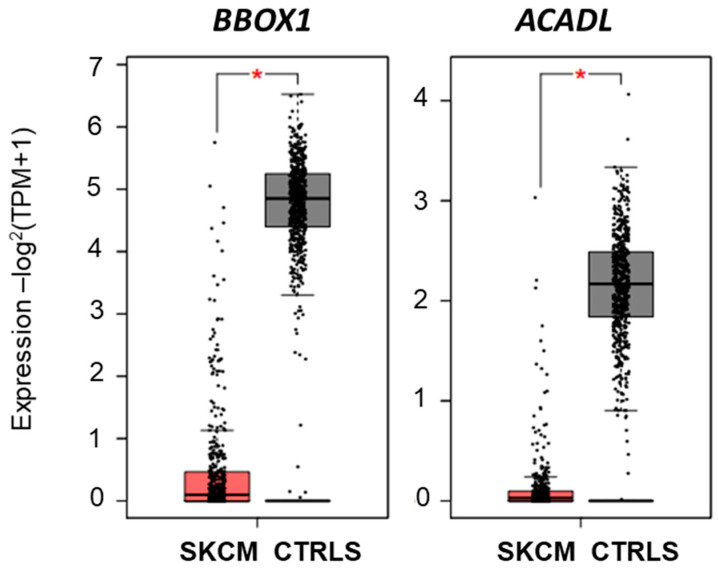
Expression levels of *BBOX1* and *ACDL* genes in 461 melanoma patients (SKCM) and 561 controls (CTRLS), according to TCGA/GTEx data available at GEPIA2 platform (http://gepia2.cancer-pku.cn/#index, accessed on 10 November 2023). * indicates *p* > 0.0001.

According to the metabolic effects reported in Table 1, several carnitine derivatives appear to be strongly affected in A375 exposed to 6 h miconazole 30 μM. Lines 1, 15, 18, 30, 38, 51, 55, 67, 78, 79 and 81 in Table 1 report significant alterations of carnitines expression or carnitine-derivatives ratios. We therefore hypothesized that carnitine metabolic balance may play a role in melanoma. The expression of 29 genes involved in carnitine metabolism was then investigated in 461 melanoma patients and 558 controls, in GEPIA2 platform (http://gepia2.cancer-pku.cn/#index, accessed on 10 November 2023). Table 2 shows that 12 carnitine-metabolism related genes are significantly modified in melanoma patients vs. ctrl; namely, two genes involved in carnitine synthesis (*SHMT2*, *BBOX1*), two carnitine carriers (*SLC22A5* and *SLC25A29*), two genes related to Carnitine Palmitoyltransferases (*CPT1B* and *ACACB*), the Carnitine O-Octanoyltransferase (*CROT*), four genes related to acethylcarnitine metabolism (*ACADM*, *ACAD8*, *ACADVL* and *ACADL*) and one gene related to carnitine deficiency (*IFT81*). Particularly impressive is the expression lowered to almost zero, in melanoma patients, for *BBOX1* and *ACADL* genes (Figure 9), never reported before to this extent. The strong expression reduction was validated in data from GENT2 database. Table 2 also shows that 6 out of 29 genes show a significant relation with melanoma patients’ survival, namely, *SHMT2*, *SLC22A4*, *CPT1B*, *CHKB*-*CPT1B*, *CRAT*, *HADHA*.

**Figure 10:**
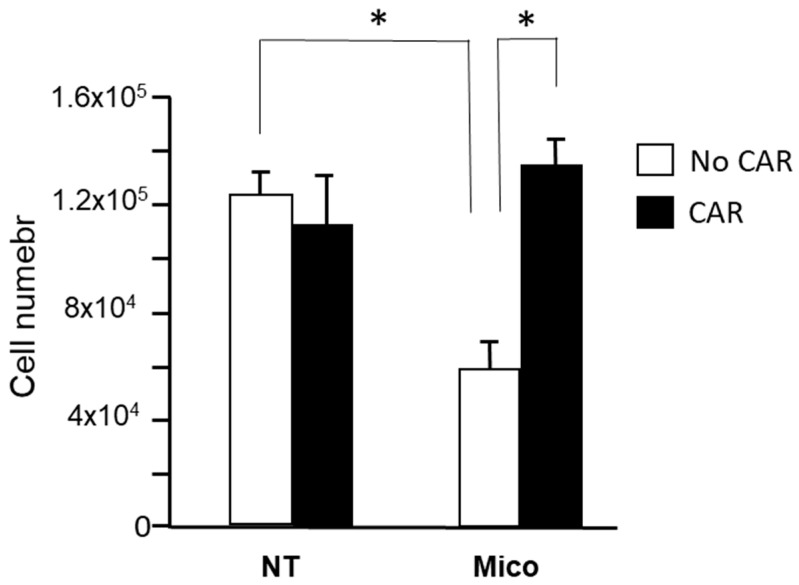
A375 proliferation (24 h) in the presence of miconazole (Mico) 30 μM, with or without carnitine (CAR) 100 μM. The inhibitory effect of miconazole is completely reversed in the presence of carnitine. NT = DMSO-treated * indicates *p* < 0.001.

Such data suggested that carnitine may play a role in the miconazole antimelanoma effect. To investigate this hypothesis, 24 h proliferation of A375 melanoma cells was measured in the presence of miconazole (30 μM), with or without carnitine (100 μM). Figure 10 shows that miconazole antiproliferation effect on A375 melanoma cells is completely reversed in the presence of carnitine, demonstrating that miconazole antimelanoma effects are, at least in part, dependent on carnitine levels.

#### 2.2.5. Investigating Pro-Apoptotic Caspases, Cell Death and Apoptosis

**Figure 11:**
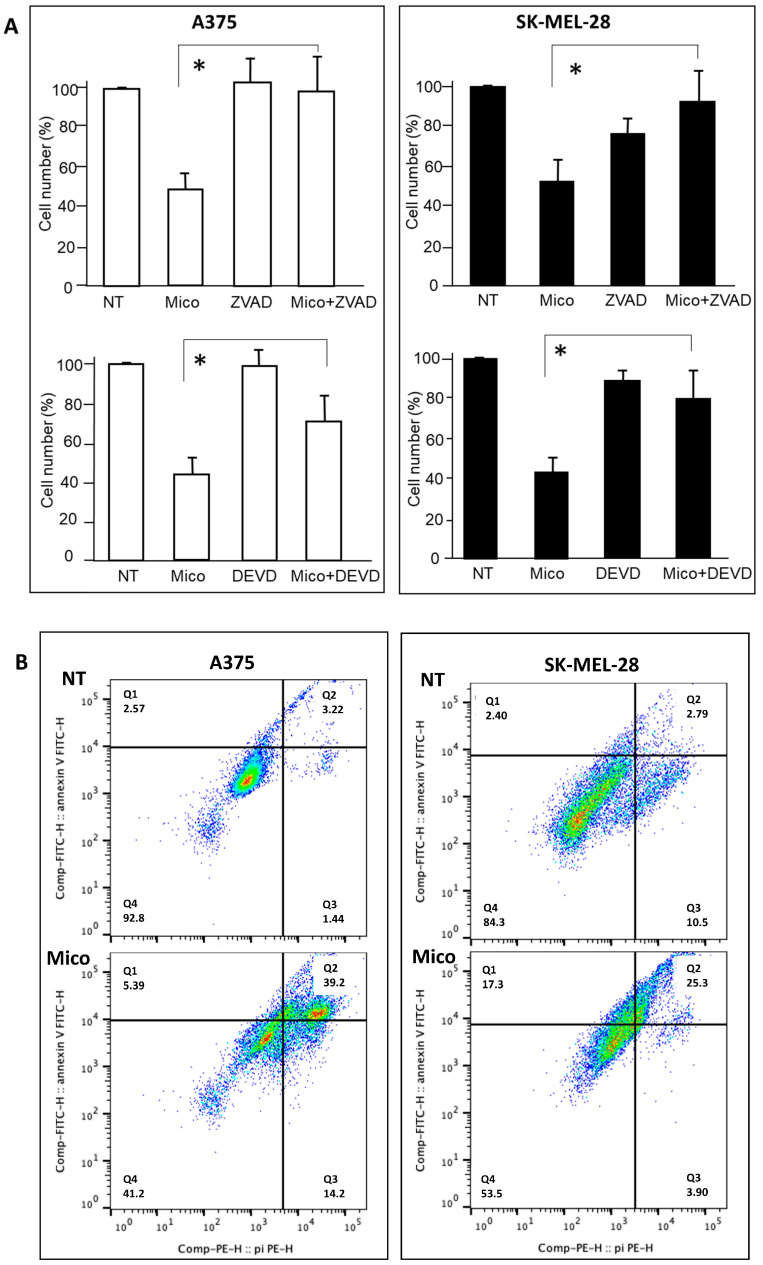
(**A**): Proliferation of A375 (left panels) and SK-MEL-28 (right panels) for 24 h, in the presence of miconazole (Mico) 30 μM, either with ZVAD (top panels) or with DEVD (bottom panels) 45 μM. The inhibitory effect of miconazole is significantly and completely reversed in the presence of ZVAD and significantly and partially reversed in the presence of DEVD. NT = DMSO-treated. * indicates *p* < 0.001. (**B**): FACS analysis of A375 and SK-MEL-28 untreated (NT = DMSO treated) or treated with miconazole, stained with FITC-conjugated Annexin V and Propidium Iodide, shows a strong increase in both early and late apoptosis, as well as necrosis (Q1, Q2 and Q3 areas, respectively). Experiments reported in panel (**B**) were performed at least in triplicate, and the images refer to a representative experiment.

In order to assess the role of pro-apoptotic caspases in the observed effects, we carried out proliferation experiments, exposing A375 and SK-MEL-28 cells to miconazole 30 μM, for 24 h, in the presence or in the absence of caspases-inhibitors such as the pan-caspases inhibitor ZVAD-FMK and the caspase 3/7 inhibitor Ac-DEVD-CHO. As depicted in Figure 11A, the miconazole effect is significantly reverted by both ZVAD and DEVD, indicating that apoptotic caspases activity, likely caspase 3 or caspase 7, is required to observe the antiproliferative effect of miconazole.

In addition, as reported in Figure 11B, both early and late apoptosis as well as necrosis are increased in A375 and in SK-MEL-28, upon miconazole 30 μM treatment for 24 h, as compared to ctrl (NT = DMSO-treated). The experiments, performed at least in triplicate, indicate that in A375 cells, early apoptosis (Q1 area) increases from 1.9 to 4.5% (*p* = 0.03), late apoptosis (Q2 area) from 2.3 to 37.7% (*p* = 0.001), and necrosis (Q3 area) increases from 2.8 to 20.9% (*p* < 0.001). A375 images reported in Figure 11B refer to a representative experiment. In SK-MEL-28 cells, miconazole treatment increases early apoptosis from 5.3 to 13.6% (*p* = 0.02) and increases late apoptosis from 3 to 25.5% (*p* = 0.03), while necrotic cells are unchanged (*p* = n.s.). SK-MEL-28 images reported in Figure 11B refer to a representative experiment. Figure 11 indicates that miconazole antimelanoma effect is strongly dependent on the activity of proapoptotic caspases (panel A) and strongly increases the number of cells in early and late apoptosis (panel B).

## 3. Discussion

Miconazole is a drug largely used in clinics, mostly for its antimycotic activity. It has shown anti-inflammatory [37] as well as antitumor effects in different cancer setups such as hepato-cellular carcinoma cells [38], bladder cancer cells [39] and breast cancer [40]. In a glioblastoma setup, miconazole was shown to induce autophagic cell death by inducing ROS production [41]. Similarly, autophagy induction by miconazole treatment has been shown in bladder cancer cells [42]. Melanoma has been poorly investigated under this respect. In a study published in 2019, we demonstrated for the first time that miconazole may exert a strong antimelanoma effect in vitro; we related such an effect, at least in part, to the expression of an ion channel, namely KCNN2 (28). Miconazole antimelanoma effect was then confirmed in a zebrafish model study published in 2020 [43], and miconazole nanoemulsions are under investigation as a possible formulation [44]. Mechanisms underlying the strong miconazole antimelanoma effect are still to be elucidated. In the present study, we show biological effects at 24 h treatments with both miconazole and clotrimazole, namely, significant inhibition of in vitro proliferation, viability and vascular mimicry (Figure 1, Figure 2 and Figure 3, respectively). The underlying mechanisms were addressed by measuring effects at 6 h time point to investigate early molecular effects likely inducing the late biological effects observed at 24 h. At this early time point, ATP production, i.e., a hallmark of mitochondrial function, was significantly reduced in a dose-dependent way by miconazole in either A375 and SK-MEL-28 melanoma cells and by clotrimazole in A375 cells (Figure 4). Further, ROS production was increased on both cell lines by miconazole (Figure 5). ATP and ROS are strongly related to mitochondria activity. In fact, ATP production occurs in mitochondria, mainly through oxidative phosphorylation [45], and 90% of ROS is produced in mitochondria [46]. Mitochondria were then the most interesting candidates to be further investigated, as possible targets of miconazole activity in melanoma cells, also according to previous data reported in bladder cancer [47]. Indeed, both whole fluorescence and single-cells fluorescence by confocal microscopy were found reduced (Figure 6, Figure 7 and Figure 8). The observed reduction in ATP production, the increase in ROS levels and the parallel reduction in mitochondria-associated fluorescence are all indicators of a strong effect on mitochondrial function. The current study also presents for the first time several evidences indicating a strong metabolic effect of miconazole on human melanoma cells, at a very early time point such as 6 h treatment. Accordingly, Table 1 shows that mitochondria-related metabolites and metabolism indicators are significantly modified in cells treated with miconazole 30 μM for 6 h. Mostly carnitines (i.e., a lysine and methionine derivatives), nearly all amino acids and amino-acids-related molecules and choline-related molecules are strongly modified, suggesting a marked early effect of miconazole on energy production metabolic effects. Particularly, carnitines levels appear strongly modified both in terms of relative ratios and as absolute values. In fact, the most modified metabolite/indicator (see line n. 1 in Table 1) is C4/C3 ratio, (i.e., Butyrylcarnitine/Propionylcarnitine ratio), increased by 12 times in 6 h miconazole-treated cells. This indicator is reported as “Short-Chain Acyl-Coenzyme A Dehydrogenase (SCAD) Deficiency (NBS) and refers to deficiency of short-chain acyl-CoA dehydrogenase, which prevents the body from converting short-chain fatty acids into energy. Carnitines were, along with amino-acids and phophatidylcholines, the molecules most profoundly modified by miconazole treatment, according to Table 1. The third most modified metabolite/indicator (i.e., n. 3 in Table 1) is C4/C2 ratio (Butyrylcarnitine/Acetylcarnitine ratio), which is increased by more than five times in miconazole treated cells at 6 h. This increase indicates isobutyryl-CoA dehydrogenase deficiency, a condition that disrupts the breakdown of the amino acid valine. Carnitine metabolism is also shown to be altered at lines n. 15, 30, 38, 67, 78 and 81 in Table 1. Carnitines metabolism is mostly at the mitochondrial level and is crucial for the fatty acid oxidation and energy production [48,49,50,51], and carnitines are reported as key mitochondrial players in different pathologic conditions [50,52,53,54,55,56]. The potential role carnitine-metabolism-related genes may play in melanoma growth was then confirmed by the analysis depicted in Table 2 and Figure 9, indicating significant expression change and significant correlation with survival in melanoma patients. Dietary intake of carnitine was previously indicated to reduce melanoma growth in mice [57] by using food dietary supplements containing several other constituents in addition to carnitine, such as coenzyme Q10, branched-chain amino acids, vitamins and zinc. The present study shows, for the first time, a strong alteration of expression of several genes related to carnitine metabolism in melanoma patients. Also, carnitine shows, in the current study, the ability to significantly reverse the antimelanoma effect of miconazole in A375 human melanoma cells. Such data demonstrate, for the first time, that carnitine may play a specific role in the mechanism of action of drugs controlling melanoma cells proliferation. The second most modified metabolite/indicator (i.e., line n. 2 in Table 1) is Lac/H1, indicating Lactate Dehydrogenase Activity, increased by more than five times in 6 h miconazole-treated cells. This is a fermentation indicator, related to pyruvate reduction by lactate dehydrogenase into lactate; this action is strongly affected in cancer cells, particularly in melanoma [58]. Its strong increase in the experimental conditions of the present study was mostly related to the relevant reduction in hexose levels (see line 69 in Table 1). Metabolites/indicators n. 4, 5 and 7 in Table 1 are asparagine synthesis, putrescine synthesis and polyamine synthesis, all strongly increased in 6 h miconazole-treated cells. Polyamine, putrescin and asparagine metabolism are known to be strongly altered in cancer [59,60,61]. Metabolism of amino acids, polyamines, ornithine and taurine are all strongly inter-connected, and their expression was in fact found significantly altered in Table 1 (see lines n. 6, 8, 18, 19, 20, 23, 35, 39, 41, 42, 52, 54, 59 and 79) indicating a profound metabolism alteration of all amino acids and proteins. Finally, choline-related metabolism was found modified in Table 1 (see line n. 16, 57, 58, 60, 61, 63, 64, 65, 66 70, 71, 73, 74, 75 and 76), indicating a significant effect of miconazole on an essential molecule in phospholipids structure as well as in neurotransmitters, linked to betaine metabolism [62] and to fatty acids synthesis, which were in fact significantly modified in Table 1 at lines n. 12, 17, 38, 51, 82.

Finally, a significant and relevant reversion of the antiproliferation effect of miconazole was observed in the presence of ZVAD-FMK (i.e., a pan-caspases-inhibitor) and DEVD Ac-DEVD-CHO (i.e., a caspase 3/7 inhibitor) in both A375 and SK-MEL-28 cells. This suggested that the miconazole effect in human melanoma cells is, at least in part, dependent on proapoptotic signals mediated by caspases activation. Further, annexin/propidium iodide staining evaluated with FACS analysis indicates a strong increase in both early and late apoptosis upon miconazole 30 μM treatment for 24 h. Under this respect, differences between A375 and SK-MEL-28 may depend on their different growth kinetics. A mitochondrial-dependent apoptosis mediated by caspase3/7 is well known in several pathogenic setups [63,64,65,66], further supporting the role mitochondria play in the antiproliferative effects reported in the present study. Therefore, the collected evidences indicate that miconazole induces apoptosis in melanoma, likely via an early mitochondria disregulation evident at 6 h treatment.

In conclusion, as recently highlighted [67], drugs with antifungal activity have potential application in cancer therapy, thanks to common molecular targets overlapping cancer targets. Namely, previous evidences suggest the possible use of miconazole in colon, bladder, breast and lung cancers and osteosarcoma. Data reported in the present study support the potential use of miconazole in melanoma.

## 4. Materials and Methods

The aim of the present study was to investigate biological effects and molecular mechanisms involved in the response of human melanoma cell lines exposed to miconazole and clotrimazole. Late biological effects (namely, cell proliferation, cell viability, vascular mimicry) were assayed at 24 h, while early molecular mechanisms (ROS release, ATP release, mitochondria staining, metabolomics profiling) were assayed at 6 h treatment.

### 4.1. Cell Culture

Human melanoma cell lines SK-MEL-28 and A375 were purchased from the American Type Culture Collection (ATCC, Manassas, VA, USA). SK-MEL-28 and A375 were grown, respectively, in Minimum Essential medium Eagle (MEM; Hyclone, South Logan, UT, USA) and Dulbecco’s modified Eagle’s medium (DMEM; Hyclone) supplemented with 10% fetal bovine serum (FBS; Hyclone), 2 mM L-glutamine and 100 mM penicillin/streptomycin (Invitrogen, Carlsbad, CA, USA) at 37 °C with 5% CO_2_. Miconazole nitrate was from CliniSciences (Paris, France) and was diluted in DMSO at 10 mM mother solution, as previously reported [28]. Clotrimazole was from Sigma Aldrich (St. Louis, MO, USA) and was diluted in DMSO at 25 mM, mother solution.

### 4.2. Treatment for 24 h: Cell Proliferation, Cell Viability, Vascular Mimicry

#### 4.2.1. Cell Proliferation

Miconazole and Clotrimazole effects were tested on 10% FBS-induced proliferation. SK-MEL-28 were plated at 1 × 10^5^ cells/plate, and A375 cells were plated at 8 × 10^4^ cells/plate in p35 Petri dishes at time 0. After 24 h, cells were starved for 18 h in serum-free medium and then treated with miconazole or clotrimazole increasing doses (up to 50 μM), in a medium containing FBS 10%.

At 24 h, cells were then harvested with 0.25% trypsin, 2.21 mM EDTA, 1× sodium bicarbonate (Corning, Manassas, VA, USA) and counted in hemocytometer.

A375 cells proliferation was also investigated in the presence of miconazole 30 μM, with or without L-carnitine 100 μM (catalog # C0158, (Sigma-Aldrich, St. Louis, MO, USA). Cells were seeded as reported above, and L-carnitine was given along with miconazole, after 6 h pre-treatment.

#### 4.2.2. MTT Cell Viability Assay

SK-MEL-28 and A375 cells were plated at 8 × 10^3^ cell/plate into a 96-well culture plate and grown in MEM or DMEM, respectively, supplemented with 10% FBS. After 24 h, cells were starved for 18 h in serum-free medium and treated with miconazole or clotrimazole (up to 100 μM) in complete fresh medium containing FBS 10%. Cell viability was measured using MTT reagent (10 × 5 mg/mL Thiazolyl Blue Tetrazolium Bromide Sigma Aldrich (St. Louis, MO, USA) dissolved in PBS. On the day of measurement, medium was replaced with serum-free media plus MTT reagent (1×) and incubated for 4 h at 37 °C. After solubilization, formazan crystals were dissolved in DMSO 100 μL solution and incubated for 15 min [34]. The formazan absorbance was quantified by measuring the light absorbance at 570 nm using the Ensight fluorometer (Perkin Elmer, Inc., Beaconsfield, UK).

#### 4.2.3. Vasculogenic Mimicry Assay

SK-MEL-28 were plated at 1 × 10^5^ cells/plate in p35 Petri dishes at time 0 and then starved for 18 h in serum-free medium. Twenty-four-well plates were coated with cold Matrigel (100 μL/well) and kept at 37 °C for 1 h until the gel solidified. Cells were treated with miconazole or clotrimazole (up to 50 μM), in the presence of 10% FBS. The formation of vessel-like channels was analyzed 24 h after plating. Images were then captured under S 40/0.45 LEICA microscope. The vessel-like formations were counted as the total number of closed polygons in random microscope fields, according to a methodology previously published [68]. Eight fields/well were assessed, and three experiments in triplicate were performed.

### 4.3. Treatment for 6 h: ROS Release, ATP Release, Mitochondria Staining, Metabolomics Profiling

#### 4.3.1. Evaluation of ROS and ATP Production

SK-MEL-28 and A375 cells were plated at 1 × 10^5^ cells/plate and 8 × 10^4^ cell/plate, respectively, in p35 Petri dishes at time 0 and then starved for 18 h in serum-free medium. After 6 h treatment with miconazole or clotrimazole (up to 50 μM) in the presence of 10% FBS, cells were washed with PBS, harvested with 0.25% trypsin and counted. Intracellular ATP content was measured by using the ATP Colorimetric/Fluorometric Assay Kit (BioVision). According to the manufacturer’s instructions, the cell lysate absorbance was measured at 450 nm, and ATP concentration was expressed in nmol/10^6^ cells.

Reactive Oxygen Species (ROS) level was measured using the 2′,7′-dichlorodihydrofluorescein diacetate (DCFDA)-Cellular Reactive Oxygen Species (ROS) Detection Assay Kit (AbCam ab 113,851). A375 and SK-MEL-28 cells were incubated with 25 μM DCFDA for 45 min at 37 °C and then treated with increasing doses of miconazole and clotrimazole in complete fresh medium for 6 h. Tert-Butyl Hydrogen Peroxide (TBHP) solution was used as a positive control for ROS production. The fluorescence intensity of controls and of treated wells was measured at the Ensight fluorometer (Perkin Elmer, Inc., Beaconsfield, UK) at Ex = 485 nm and Em = 535 nm, according to the manufacturer’s instructions as previously reported [35].

#### 4.3.2. Mitochondria Investigation: Whole Fluorescence

SK-MEL-28 and A375 cells were plated at 8 × 10^3^ cell/plate into 96-well culture plates and grown in MEM or DMEM supplemented with 10% FBS. Cells were then starved for 18 h in serum-free medium and then treated with miconazole (up to 50 μM) dissolved in complete fresh medium containing 10% FBS. After 6 h treatment at 37 °C in 96 wells plates, cells were incubated with 10 nM Biotracker™ 488 Green Mitochondria Dye (Merck Millipore Bio, Temecula, CA, USA) for 15 min at 37 °C according to manufacturer instructions. The mitochondria fluorescence intensity of treated and untreated cells was then measured at the Ensight fluorometer (Perkin Elmer, Inc., Beaconsfield, UK) at Ex = 490 nm and Em = 523 nm, detected using the green FITC/GFP channel.

#### 4.3.3. Mitochondria Investigation: Confocal Cytofluorescence

SK-MEL-28 cells (1 × 10^5^) were seeded on sterile glass slides (20 × 20 mm) placed in six-multiwells plates. Cells were kept for 24 h in medium without serum, then the medium was substituted with medium-10% serum + DMSO (ctrl) or medium-10% serum + Miconazole 50 μM in DMSO and treated for 6 h. Cells were then fixed with formaldehyde 3.7% for 15 min at RT, washed two times in blocking buffer BSA 1% PBS (Bovine Serum Albumin, Sigma Aldrich, Darmstadt, Germany) to minimize a-specific binding. Cells were then incubated with BioTracker 488 Green Mitochondria dye (Merck Millipore Bio, Temecula, CA, USA) 20 μM for 30 min at 37 °C. Finally, cover slide was mounted with Vectashield (ref. H1200, Vector Laboratories, Burlingame, CA, USA). Cytofluorescence images under confocal microscope (LSM 510, Zeiss, Oberkochen, Baden-Württemberg, Germany) were taken. Images were obtained in scan mode by frame, size 512 × 512 nm^2^, Pixel Dwell 25.00 µs, revealed by Argon/2 laser at 488 nm wavelength. Images were then analyzed with the data processing software ZEN core 3.8 (ZEISS, Oberkochen, Baden-Württemberg, Germany) and with “Images J2” with Java 8 (NHI, LOCI, University of Wisconsin, Madison, WI, USA). Mean fluorescence intensity was calculated and normalized by nuclei number, in 30 fields for sample. In more detail: by using the tool “Measure” of ImageJ (version 2.14.0/1.54F), the mean fluorescence intensity, after subtracting the background, was calculated for each field. Data were then normalized for the number of nuclei in each field. Mean, standard deviation and *t*-test analysis were then performed for treated and untreated samples.

Mitochondria fluorescence was then investigated with the “Mitochondrial analyzer” Fiji plugin, downloaded at https://github.com/AhsenChaudhry/Mitochondria-Analyzer (accessed on 10 March 2024), able to perform a mitochondrial specific analysis, as previously reported [69,70]. In more detail, the image analyzer performs an adaptative thresholding of the image for the correct extraction of mitochondrial objects. After a pre-processing noise optimization step, the software makes background subtraction, sigma filter and local contrast enhancement. This procedure extracted a clean picture of mitochondria (Figure 7C,D) from the images of Figure 7A,B.

#### 4.3.4. Metabolomics Analysis

Metabolomics analyses were carried out on A375 cells, untreated or treated with miconazole for 6 h. Cells were plated at 1 × 10^6^ cells/plate, starved for 18 h in serum-free medium and then treated with miconazole 30 μM in medium containing FBS 10%. After 6 h treatment, the cell medium was removed, and cells were quickly rinsed with pre-cooled 0.9% NaCl solution. Extraction was performed on ice by adding 180 µL of ice-cold isopropanol per 1 × 10^6^ cells and quickly detached by scraping, according to Biocrates instructions (Biocrates, Innsbruck, Austria) with minor modifications. Extracts were transferred to 1.5 mL microcentrifuge tubes, immediately frozen, and then sent to Biocrates for analysis. Analytical methods were applied, according to Biocrates (Biocrates, Innsbruck, Austria). Briefly: the commercially available MxP^®^ Quant 500 XL kit from Biocrates was used for the quantification of several endogenous metabolites of various biochemical classes. Lipids and hexoses were measured by flow injection analysis-tandem mass spectrometry (FIA-MS/MS) using a 5500 QTRAP^®^ instrument (AB Sciex, Darmstadt, Germany) with an electrospray ionization (ESI) source, and small molecules were measured by liquid chromatography-tandem mass spectrometry (LC-MS/MS) using a different 5500 QTRAP^®^ instrument of the same type. The extracts were analyzed by FIA-MS/MS and LC-MS/MS methods using multiple reaction monitoring (MRM). For the XL-part, a predefined amount of underivatized samples was pipetted on an additional plate with inserts carrying lipid internal standards. LC concentrations were calculated using appropriate mass spectrometry software (Sciex Analyst^®^, https://biocrates.com/wp-content/uploads/2023/06/Application-note-Quant500-XL-across-MS-platforms.pdf, accessed on 10 March 2024) and FIA-concentrations were quantified using the Biocrates WebIDQ software (https://biocrates.com/webidq/, accessed on 10 March 2024). The assay has been validated based on the European Medicines Agency (EMA) guidelines. All concentration values were normalized to external target values of Biocrates quality controls that are routinely run on every plate.

Under our experimental conditions, the cell number in treated samples was indicated to be halved at 24 h and un-modified at 6 h treatments, vs. T0.

### 4.4. Analysis of Carnitine Role

Genes related to carnitine metabolism were identified from the Human Gene database available at the Genecards platform (https://www.genecards.org/, accessed on 10 March 2024); their expression was then analyzed with the “Box plot” tool at the GEPIA2 platform/http://gepia2.cancer-pku.cn/#analysis, accessed on 10 March 2024). Expression data were then validated on GENT2 platform (http://gent2.appex.kr/gent2/, accessed on 10 March 2024).

In addition, A375 cells proliferation was investigated in the presence of miconazole 30 μM, with or without L-carnitine 100 μM. Cells were seeded as reported before; miconazole, and L-carnitine was added alone or with miconazole, after 6 h pre-treatment

### 4.5. Apoptosis Analyses

The role of proapoptotic caspases activation was investigated by exposing A375 and SK-MEL-28 cells to miconazole 30 μM, for 24 h, in the presence or in the absence of caspases-inhibitors such as the pan-caspases inhibitor ZVAD-FMK (MCE Medicalchem express catalog. N. HY16658B 1 Deer Park Dr, Suite Q, Monmouth Junction, NJ 08852, USA) and the caspase 3/7 inhibitor Ac-DEVD-CHO (Euroclone S.p.A; catalog n. ALX-260-030-M001; Enzo Life Sciences Inc., Via Figino, 20/22 20016 Pero, MI, Italy). Both inhibitors were used at 45 μM concentration. A375 as SK-MEL-28 cells, untreated (i.e., treated with DMSO only) or treated with miconazole 30 μM for 24 h, were washed twice in PBS, resuspended in Annexin binding buffer and stained with FITC- conjugated Annexin V and Propidium Iodide (PI), according to the manufacturer’s instruction. The Annexin V-FITC Apoptosis detection Kit was used, catalog n. BMS500FI/20 from Bender MedSystems GmbH Campus Vienna Biocenter 2 1030 Vienna, Austria. FACS analysis was performed using BD FACSMelody™ Cell Sorter (Beckman Coulter Life Sciences, Indianapolis, IN, USA) using channel 488 for FITC-Conjugated Annexin V and filter PE with range 610–630 nm for PI. Data were collected in duplicate from five different experiments and further analyzed with FlowJo 10 software (Beckman Coulter Life Sciences, 385 Williamson Way Ashland, OR 97520, USA).

### 4.6. Statistical Analyses

Data were expressed as mean ± S.D. from at least three independent experiments. Statistical analysis was performed using Student’s *t*-test or one-way ANOVA test or Friedman test, when indicated. *p* value ≤ 0.05 was considered the statistically significant threshold. Statistical analyses were performed on GraphPad Prism 9 (GraphPad Software Inc., Boston, MA, USA). Statistical analyses of metabolomics data: to enable a comparison of the concentrations in different groups, all values were normalized to the cell number, i.e., 2 × 10^6^ at T0, 2 × 10^6^ at 6 h in treated samples, and 1 × 10^6^ cell number at 24 h in treated samples. Amounts were then expressed as pmol/10^6^ cells. The analysis involved the quantification of 1019 metabolites; 576 analytes were eliminated from the statistical analysis since their concentration was missing or below the detection limit. The following analysis was then carried out on 443 analytes. Concentration was measured and log10 transformation was achieved. Then, ANOVA analysis was carried out on the groups. The delta for the log10-transformed values was obtained; to get the ratio between the concentrations, this delta was reversed. In the present study, the effect measured on the group “6 h in DMSO” was compared to the group “6 h in DMSO + Miconazole 30 μM”, to focus on early effects. The multiple testing correction was performed according to Benjamini procedure [36]. It adjusts the *p* value to take the many comparisons (i.e., one for each metabolite) into account, resulting in a q value (=FDR-adjusted *p* value). Metabolites concentration was then considered statistically different only with *p* < 0.05 AND q < 0.2. Out of 443 analyzed metabolites, 83 metabolites/indicators were found significantly modified at 6 h DMSO + miconazole treatment vs. DMSO treated.

## 5. Conclusions

Miconazole potently interferes in mitochondrial activity in melanoma cells, leading to a strong caspases-dependent antimelanoma activity. Such effects depend at least in part on carnitine and may suggest miconazole use within a novel antimelanoma strategy.

## Figures and Tables

**Table 1:** Metabolites found significantly modified in A375 cells treated with miconazole for 6 h. Values are the ratio of treatment vs. DMSO. Values > 1 indicate increase in miconazole-treated samples, while <1 indicates reduction in miconazole-treated samples. Numbers are sorted by ratio value. Significance was measured according to ANOVA analysis.

N	Metabolite or Metabolite Indicator	Ratio	*p* Value	Formula
1	Short-Chain Acyl-Coenzyme A Dehydrogenase (SCAD) Deficiency (NBS)	12.46	3.3^−13^	C4/C3
2	Lactate Dehydrogenase Activity	5.39	1.3^−7^	Lac/H1
3	Isobutyryl-Coenzyme A Dehydrogenase Deficiency (NBS)	5.37	2.6^−11^	C4/C2
4	Monoglyceride 16:1	5.16	0.005	
5	Asparagine Synthesis	3.33	5.0^−7^	Asn/Asp
6	Putrescine Synthesis	2.87	0.007	Putrescine/Orn
7	Lysophosphatidyl-choline 17:0	2.67	0.018	
8	Polyamine Synthesis	2.45	0.023	(Putrescine + Spermidine + Spermine)/Orn
9	Sarcosine Synthesis from Choline	2.37	0.002	Sarcosine/Choline
10	Glutaminolysis Rate	2.24	8.5^−6^	(Ala + Asp + Glu + Lac + Suc)/Gln
11	Lysophosphatidyl-glycerol 16:1	2.07	4.6^−4^	
12	Betaine Synthesis	2.01	0.004	Betaine/Choline
13	Asparagine	1.96	0.012	
14	Phosphatidyl-ethanolamine P-16:0/15:0	1.95	0.008	
15	Butyrylcarnitine	1.84	0.002	
16	Lysophosphatidyl-choline 16:0	1.81	0.031	
17	Sphingosine phosphate d18:1	1.73	4.0^−4^	
18	2-Methylbutyrylglycinuria (NBS)	1.69	0.011	C5/C3
19	Taurine Synthesis	1.67	0.025	Taurine/Cys
20	Gamma-Aminobutyric Acid Synthesis	1.47	0.005	GABA/Glu
21	Methionine Oxidation	0.85	0.017	Met-SO/Met
22	Glycine Synthesis	0.80	1.6^−4^	Gly/Ser
23	Dihydrolipoamide Dehydrogenase Deficiency (NBS)	0.71	0.008	Pro/Phe
24	5-Aminovaleric acid	0.70	0.017	
25	beta-Alanine	0.69	0.025	
26	Hydroxyglutaric acid	0.68	0.003	
27	Serine	0.67	0.010	
28	Alanine	0.63	0.031	
29	Taurine	0.62	0.006	
30	Carnitine	0.60	4.4^−4^	
31	Triacylglyceride 18:1_33:3	0.60	0.009	
32	Threonine	0.59	0.007	
33	Glutamine	0.59	0.002	
34	Aspartic acid	0.59	0.028	
35	Sum of Amino Acids	0.59	0.004	
36	alpha-Aminoadipic acid	0.59	0.018	
37	trans-4-Hydroxyproline	0.58	0.010	
38	Ratio of Acetylcarnitine to Carnitine	0.57	0.008	C2/C0
39	Sum of Solely Glucogenic Amino Acids	0.57	0.002	Ala + Arg + Asn + Asp + Cys + Gln + Glu + Gly + His + Met + Pro + Ser + Thr + Val
40	beta-Aminobutyric acid	0.56	0.012	
41	Sum of Non-Essential Amino Acids	0.56	8.9 × 10^−4^	Ala + Arg + Asn + Asp + Cys + Gln + Glu + Gly + Pro + Ser + Tyr
42	Cysteine Synthesis	0.55	6.0 × 10^−4^	Cys/(Ser + Met)
43	Glutamic acid	0.55	1.7 × 10^−4^	
44	Glicine	0.54	0.002	
45	Taurolithocholic acid	0.51	0.013	
46	Carnosine Synthesis	0.51	0.029	Carnosine/His
47	Glutathione Constituents	0.50	1.2 × 10^−4^	Glu + Gly + Cys
48	Proline	0.48	8.2 × 10^−4^	
49	Sum of Conjugated Primary Bile Acids	0.47	0.024	GCA + GCDCA + TCA + TCDCA
50	Taurochenodeoxy-cholic acid	0.46	0.019	
51	Beta-Oxidation	0.44	4.8 × 10^−4^	(C2 + C3)/C0
52	Sum of Taurine-Conjugated Bile Acids	0.43	0.014	TCA + TCDCA + TDCA + TLCA
53	Malonic Aciduria (NBS)	0.43	1.6 × 10^−5^	C3/C2
54	Sum of Sulfur-Containing Amino Acids	0.43	0.002	Met + Cys
55	Short/Branched-Chain Acyl-Coenzyme A Dehydrogenase Deficiency (NBS)	0.42	5.4 × 10^−5^	C5/C0
56	Taurodeoxycholic acid	0.41	0.016	
57	Phosphatidyl-choline O-38:3	0.39	0.023	
58	Phosphatidyl-choline O-36:5	0.38	0.024	
59	Ornithine	0.38	0.031	
60	Phosphatidyl-choline O-36:3	0.38	0.020	
61	Phosphatidyl-choline O-38:6	0.38	0.018	
62	Cysteine	0.37	7.8 × 10^−4^	
63	Choline	0.37	6.3 × 10^−4^	
64	Phosphatidyl-choline 36:3	0.35	0.02	
65	Phosphatidyl-choline 38:4	0.35	0.022	
66	Phosphatidyl-choline 40:6	0.35	0.027	
67	Acetylcarnitine	0.34	3.9 × 10^−5^	
68	Aconitic acid	0.33	5.0 × 10^−4^	
69	Hexose	0.32	0.013	
70	Phosphatidyl-choline 38:5	0.32	0.011	
71	Phosphatidyl-choline O-36:4	0.32	0.008	
72	Taurocholic acid	0.32	0.005	
73	Phosphatidyl-choline 36:4	0.31	0.009	
74	Phosphatidyl-choline 36:5	0.31	0.008	
75	Phosphatidyl-choline O-38:4	0.31	0.007	
76	Phosphatidyl-choline 38:6	0.29	0.007	
77	Succinic acid	0.26	2.5 × 10^−4^	
78	Valerylcarnitine	0.25	7.8 × 10^−7^	C5
79	Methylmalonic Acidemia (NBS)	0.25	1.8 × 10^−5^	C3/C0
80	Sphinganine d14:0	0.21	0.004	
81	Propionylcarnitine	0.15	8.6 × 10^−7^	C3
82	Triacylglyceride 18:1_33:0	0.07	0.004	
83	Phosphatidyl-inositol (18:1_20:2)	0.04	0.011	

**Table 2:** Expression of carnitine metabolism-related genes in melanoma vs. ctrls, and the relation of their expression with patients’ survival (n.s. = not significant change).

Enzymes Related to Carnitine Metabolism	Expression in Melanoma vs. Ctrls log_2_(TPM-1)	Patients’ Survival Hazard Ratio (*p* < 0.05)
**Related to carnitine synthesis**		
TMLD	n.s.	n.s.
TMABADH	n.s	n.s.
SHMT1	n.s	n.s.
SHMT2	6.8 vs. 5.5 *	HR 1.4 (*p* = 0.02)
BBOX1	0.1 vs. 4.9 *	n.s.
ALDH9A1	n.s.	n.s.
TMLHE	n.s.	n.s.
ALDH9A1	n.s.	n.s.
**Carnitine Carriers**		
SLC22A5	2.1 vs. 3.5 *	n.s.
SLC25A20	n.s.	n.s.
SLC22A4	n.s.	HR 0.66 (*p* = 0.002)
SLC22A16	n.s.	n.s.
SLC25A29	3.9 vs. 5.4 *	n.s.
SLC16A9	n.s.	n.s.
**Related to Carnitine Palmitoyltransferases**		
CPT2	n.s.	n.s.
CPT1A	n.s.	n.s.
CPT1B	3 vs. 4.2 *	HR 0.74 (*p* = 0.02)
CPT1C	n.s.	n.s.
ACACB	2.1 vs. 3.9 *	n.s.
CHKB-CPT1B	n.s.	HR 0.75 (*p* = 0.03)
**Carnitine O-Acetyltransferase**		
CRAT	n.s.	HR 1.3 (*p* = 0.04)
**Carnitine O-Octanoyltransferase**		
CROT	2.9 vs. 3.9 *	n.s.
**Related to acethylcanitine metabolism**		
ACADM	5 vs. 4 *	n.s.
ACAD8	3.6 vs. 5 *	n.s.
ACADS	n.s.	n.s.
ACADVL	7.5 vs. 9 *	n.s.
ACADL	0.1 vs. 2.1 *	n.s.
**Related to carnitine deficiency**		
HADHA	n.s.	HR = 1.4 (*p* = 0.009)
IFT81	3.6 vs. 2.7 *	n.s.

* *p* < 0.0001.

## Data Availability

Availability of data and materials: reagents and materials used in the present study are commercially available. The datasets generated and/or analyzed during the current study are available as Supplementary Materials Tables S1–S3 and in the Mendeley data repository, available after manuscript acceptance, at: Reserved DOI:10.17632/d8nsdsfb5b.1.

## Supplementary Materials

The following supporting information can be downloaded at: https://www.mdpi.com/article/10.3390/ijms25073589/s1.
