# Supplementary material for: Anti-Melanoma Effects of Miconazole: Investigating the Mitochondria Involvement
Source: Int J Mol Sci. 2024 Mar 22;25(7):3589. doi: 10.3390/ijms25073589 (PMC11011910; doi:10.3390/ijms25073589)
Supplement: Supplementary file 1 [file ijms-25-03589-s001.zip › ijms-2877172-supplementary.pdf]

Not shown material

Supplementary Figure S1

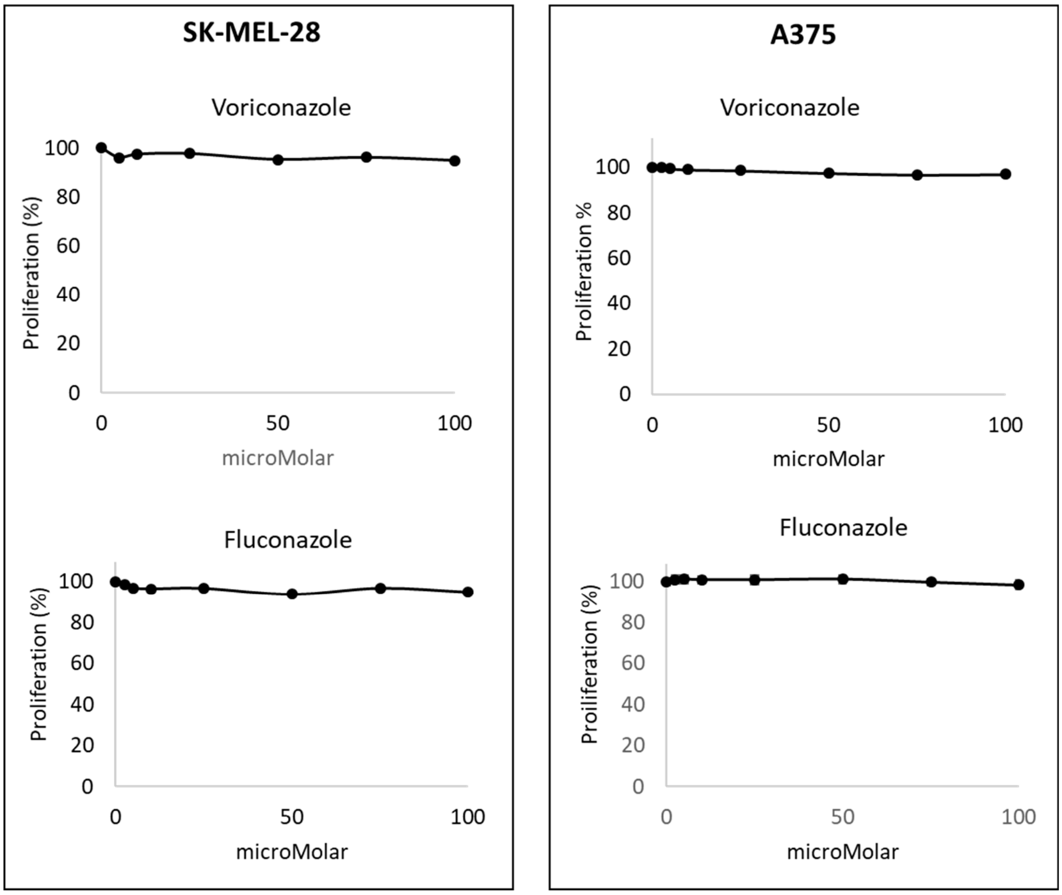

Figure S1 Supplementary. Effect of voriconazole and fluconazole on A375 and SK-MEL-28 cells proliferation, induced by serum.

Supplementary Figure S2

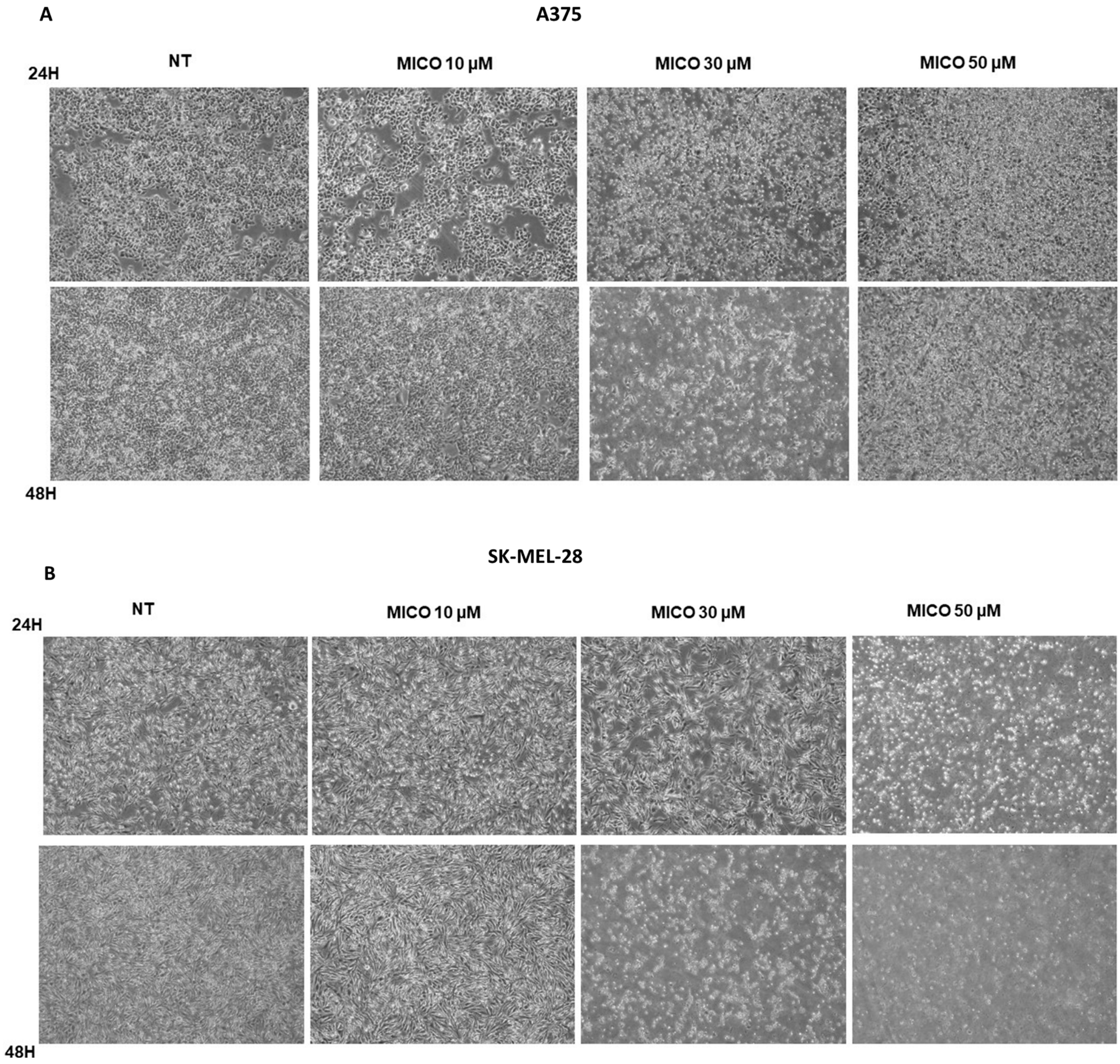

Figure S2 Supplementary. Microphotographs of A375 and SK-MEL-28 melanoma cell lines, treated with DMSO only (NT) or with increasing doses of miconazole. Magnification 10×. (A) after A375, and (B) after SK-MEL-28.

Table S1 not shown: list of 443 metabolites measured.

N.	Code	Analyte Class	Analyte Name
1	C0	Acylcarnitines	Carnitine
2	C2	Acylcarnitines	Acetylcarnitine
3	C3	Acylcarnitines	Propionylcarnitine
4	C4	Acylcarnitines	Butyrylcarnitine
5	C5	Acylcarnitines	Valerylcarnitine
6	1-Met-His	Amino acid related	1-Methylhistidine
7	3-Met-His	Amino acid related	3-Methylhistidine
8	5-AVA	Amino acid related	5-Aminovaleric acid
9	AABA	Amino acid related	alpha-Amino-butyric acid
10	ADMA	Amino acid related	Asymmetric dimethylarginine
11	alpha-AAA	Amino acid related	alpha-Aminoadipic acid
12	Anserine	Amino acid related	L-Anserine
13	BABA	Amino acid related	beta-Aminobutyric acid
14	Betaine	Amino acid related	Betaine
15	Carnosine	Amino acid related	Carnosine
16	Cit	Amino acid related	Citrulline
17	Cystine	Amino acid related	Cystine
18	HArg	Amino acid related	Homoarginine
19	HCys	Amino acid related	Homocysteine
20	Kynurenine	Amino acid related	Kynurenine
21	Met-SO	Amino acid related	Methionine-Sulfoxide
22	Orn	Amino acid related	Ornithine
23	PAG	Amino acid related	Phenylacetyl-glycine
24	ProBetaine	Amino acid related	Proline betaine
25	Sarcosine	Amino acid related	Sarcosine
26	SDMA	Amino acid related	Symmetric dimethylarginine
27	t4-OH-Pro	Amino acid related	trans-4-Hydroxyproline
28	Taurine	Amino acid related	Taurine
29	Ala	Amino acids	Alanine
30	Arg	Amino acids	Arginine
31	Asn	Amino acids	Asparagine
32	Asp	Amino acids	Aspartic acid
33	Cys	Amino acids	Cysteine
34	Gln	Amino acids	Glutamine
35	Glu	Amino acids	Glutamic acid
36	Gly	Amino acids	Glycine
37	His	Amino acids	Histidine
38	Ile	Amino acids	Isoleucine
39	Leu	Amino acids	Leucine
40	Lys	Amino acids	Lysine
41	Met	Amino acids	Methionine
42	Phe	Amino acids	Phenylalanine
43	Pro	Amino acids	Proline
44	Ser	Amino acids	Serine
45	Thr	Amino acids	Threonine
46	Trp	Amino acids	Tryptophan
47	Tyr	Amino acids	Tyrosine
48	Val	Amino acids	Valine

49	GCA	Bile acids	Glycocholic acid
50	GCDCA	Bile acids	Glycochenodeoxy-cholic acid
51	GDCA	Bile acids	Glycodeoxycholic acid
52	TCA	Bile acids	Taurocholic acid
53	TCDCA	Bile acids	Taurochenodeoxy-cholic acid
54	TDCA	Bile acids	Taurodeoxycholic acid
55	TLCA	Bile acids	Taurolithocholic acid
56	beta-Ala	Biogenic amines	beta-Alanine
57	GABA	Biogenic amines	gamma-Amino-butyric acid
58	Putrescine	Biogenic amines	Putrescine
59	Serotonin	Biogenic amines	Serotonin
60	Spermidine	Biogenic amines	Spermidine
61	Spermine	Biogenic amines	Spermine
62	Hexose	Carbohydrates and related	Hexose
63	AconAcid	Carboxylic acids	Aconitic acid
64	HipAcid	Carboxylic acids	Hippuric acid
65	Lac	Carboxylic acids	Lactic acid
66	OH-GlutAcid	Carboxylic acids	Hydroxyglutaric acid
67	Suc	Carboxylic acids	Succinic acid
68	CerP d18:1/16:0	Ceramides	Ceramide phosphate
69	Cer d18:1/14:0	Ceramides	Ceramide d18:1/14:0
70	Cer d18:1/16:0	Ceramides	Ceramide d18:1/16:0
71	Cer d18:1/18:0	Ceramides	Ceramide d18:1/18:0
72	Cer d18:1/20:0-OH	Ceramides	Ceramide d18:1/20:0-OH
73	Cer d18:1/22:0	Ceramides	Ceramide d18:1/22:0
74	Cer d18:1/24:0	Ceramides	Ceramide d18:1/24:0
75	Cer d18:1/24:1	Ceramides	Ceramide d18:1/24:1
76	Cer d18:2/16:0	Ceramides	Ceramide d18:2/16:0
77	CE 14:0	Cholesteryl esters	Cholesteryl ester 14:0
78	CE 16:0	Cholesteryl esters	Cholesteryl ester 16:0
79	CE 16:1	Cholesteryl esters	Cholesteryl ester 16:1
80	CE 18:1	Cholesteryl esters	Cholesteryl ester 18:1
81	p-Cresol-SO4	Cresols	p-Cresol sulfate
82	DG 14:0_14:0	Diglycerides	Diacylglyceride 14:0_14:0
83	DG 14:0_20:0	Diglycerides	Diacylglyceride 14:0_20:0
84	DG 16:0_16:1	Diglycerides	Diacylglyceride 16:0_16:1
85	DG 16:0_18:1	Diglycerides	Diacylglyceride 16:0_18:1
86	DG 16:1_18:1	Diglycerides	Diacylglyceride 16:1_18:1
87	DG 18:1_18:1	Diglycerides	Diacylglyceride 18:1_18:1
88	Hex2Cer d18:1/16:0	Dihexosyl-ceramides	Dihexosylceramide d18:1/16:0
89	Hex2Cer d18:1/18:0	Dihexosyl-ceramides	Dihexosylceramide d18:1/18:0
90	Hex2Cer d18:1/24:1	Dihexosyl-ceramides	Dihexosylceramide d18:1/24:1
91	FA 22:6	Fatty acids	Docosahexaenoic acid
92	Hex-Cer d18:1/14:0	Hexosylceramides	Hexosylceramide d18:1/14:0
93	Hex-Cer d18:1/16:0	Hexosylceramides	Hexosylceramide d18:1/16:0
94	Hex-Cer d18:1/18:0	Hexosylceramides	Hexosylceramide d18:1/18:0
95	Hex-Cer d18:1/20:0	Hexosylceramides	Hexosylceramide d18:1/20:0
96	Hex-Cer d18:1/22:0	Hexosylceramides	Hexosylceramide d18:1/22:0
97	Hex-Cer d18:1/23:0	Hexosylceramides	Hexosylceramide d18:1/23:0
98	Hex-Cer d18:1/24:0	Hexosylceramides	Hexosylceramide d18:1/24:0

99	Hex-Cer d18:1/24:1	Hexosylceramides	Hexosylceramide d18:1/24:1
100	Hex-Cer d18:1/26:1	Hexosylceramides	Hexosylceramide d18:1/26:1
101	Hex-Cer d18:2/16:0	Hexosylceramides	Hexosylceramide d18:2/16:0
102	Hex-Cer d18:2/18:0	Hexosylceramides	Hexosylceramide d18:2/18:0
103	Hex-Cer d18:2/22:0	Hexosylceramides	Hexosylceramide d18:2/22:0
104	Hex-Cer d18:2/24:0	Hexosylceramides	Hexosylceramide d18:2/24:0
105	3-IAA	Indoles and derivatives	Indoleacetic acid
106	LPC 16:0	Lysophosphatidyl-cholines	Lysophosphatidyl-choline 16:0
107	LPC 16:1	Lysophosphatidyl-cholines	Lysophosphatidyl-choline 16:1
108	LPC 17:0	Lysophosphatidyl-cholines	Lysophosphatidyl-choline 17:0
109	LPC 18:0	Lysophosphatidyl-cholines	Lysophosphatidyl-choline 18:0
110	LPC 18:1	Lysophosphatidyl-cholines	Lysophosphatidyl-choline 18:1
111	LPC 18:2	Lysophosphatidyl-cholines	Lysophosphatidyl-choline 18:2
112	LPC 20:4	Lysophosphatidyl-cholines	Lysophosphatidyl-choline 20:4
113	LPC 26:0	Lysophosphatidyl-cholines	Lysophosphatidyl-choline 26:0
114	LPE 14:0	Lysophosphatidyl-	Lysophosphatidyl-
115	LPE 16:0	Lysophosphatidyl-	Lysophosphatidyl-
116	LPE 16:1	Lysophosphatidyl-	Lysophosphatidyl-
117	LPE 17:1	Lysophosphatidyl-	Lysophosphatidyl-
118	LPE 18:0	Lysophosphatidyl-	Lysophosphatidyl-
119	LPE 18:1	Lysophosphatidyl-	Lysophosphatidyl-
120	LPE 18:2	Lysophosphatidyl-	Lysophosphatidyl-
121	LPE 19:1	Lysophosphatidyl-	Lysophosphatidyl-
122	LPE 20:1	Lysophosphatidyl-	Lysophosphatidyl-
123	LPE 20:2	Lysophosphatidyl-	Lysophosphatidyl-
124	LPE 20:3	Lysophosphatidyl-	Lysophosphatidyl-
125	LPE 20:4	Lysophosphatidyl-	Lysophosphatidyl-
126	LPE 20:5	Lysophosphatidyl-	Lysophosphatidyl-
127	LPE 22:1	Lysophosphatidyl-	Lysophosphatidyl-
128	LPE 22:4	Lysophosphatidyl-	Lysophosphatidyl-
129	LPE 22:5	Lysophosphatidyl-	Lysophosphatidyl-
130	LPE 22:6	Lysophosphatidyl-	Lysophosphatidyl-
131	LPE P-16:0	Lysophosphatidyl-	Lysophosphatidyl-
132	LPE P-17:0	Lysophosphatidyl-	Lysophosphatidyl-
133	LPE P-18:0	Lysophosphatidyl-	Lysophosphatidyl-
134	LPE P-18:1	Lysophosphatidyl-	Lysophosphatidyl-
135	LPE P-20:0	Lysophosphatidyl-	Lysophosphatidyl-
136	LPG 16:1	Lysophosphatidyl-glycerols	Lysophosphatidyl-glycerol
137	LPG 18:1	Lysophosphatidyl-glycerols	Lysophosphatidyl-glycerol
138	LPI 18:0	Lysophosphatidyl-inositols	Lysophosphatidyl-inositol 18:0
139	LPI 18:1	Lysophosphatidyl-inositols	Lysophosphatidyl-inositol 18:1
140	LPS 16:0	Lysophosphatidyl-serines	Lysophosphatidyl-serine 16:0
141	LPS 18:0	Lysophosphatidyl-serines	Lysophosphatidyl-serine 18:0
142	LPS 18:1	Lysophosphatidyl-serines	Lysophosphatidyl-serine 18:1
143	MG 16:1	Monoglycerides	Monoglyceride 16:1
144	MG 20:4	Monoglycerides	Monoglyceride 20:4
145	Hypoxanthine	Nucleobases and related	Hypoxanthine
146	Xanthine	Nucleobases and related	Xanthine
147	PA 17:1_18:1	Phosphatidic acids	Phosphatidic acid (17:1_18:1)
148	PA 18:0_18:1	Phosphatidic acids	Phosphatidic acid (18:0_18:1)

149	PA 18:0_18:2	Phosphatidic acids	Phosphatidic acid (18:0_18:2)
150	PA 18:1_18:1	Phosphatidic acids	Phosphatidic acid (18:1_18:1)
151	PA 18:1_20:0	Phosphatidic acids	Phosphatidic acid (18:1_20:0)
152	PA 18:1_20:1	Phosphatidic acids	Phosphatidic acid (18:1_20:1)
153	PA 18:1_20:2	Phosphatidic acids	Phosphatidic acid (18:1_20:2)
154	PA 18:2_18:3	Phosphatidic acids	Phosphatidic acid (18:2_18:3)
155	PA 18:2_20:0	Phosphatidic acids	Phosphatidic acid (18:2_20:0)
156	PA 18:2_20:1	Phosphatidic acids	Phosphatidic acid (18:2_20:1)
157	PA 20:0_20:4	Phosphatidic acids	Phosphatidic acid (20:0_20:4)
158	PC 24:0	Phosphatidyl-cholines	Phosphatidyl-choline 24:0
159	PC 28:1	Phosphatidyl-cholines	Phosphatidyl-choline 28:1
160	PC 30:0	Phosphatidyl-cholines	Phosphatidyl-choline 30:0
161	PC 30:2	Phosphatidyl-cholines	Phosphatidyl-choline 30:2
162	PC 32:0	Phosphatidyl-cholines	Phosphatidyl-choline 32:0
163	PC 32:1	Phosphatidyl-cholines	Phosphatidyl-choline 32:1
164	PC 32:2	Phosphatidyl-cholines	Phosphatidyl-choline 32:2
165	PC 32:3	Phosphatidyl-cholines	Phosphatidyl-choline 32:3
166	PC 34:1	Phosphatidyl-cholines	Phosphatidyl-choline 34:1
167	PC 34:2	Phosphatidyl-cholines	Phosphatidyl-choline 34:2
168	PC 34:3	Phosphatidyl-cholines	Phosphatidyl-choline 34:3
169	PC 34:4	Phosphatidyl-cholines	Phosphatidyl-choline 34:4
170	PC 36:0	Phosphatidyl-cholines	Phosphatidyl-choline 36:0
171	PC 36:1	Phosphatidyl-cholines	Phosphatidyl-choline 36:1
172	PC 36:2	Phosphatidyl-cholines	Phosphatidyl-choline 36:2
173	PC 36:3	Phosphatidyl-cholines	Phosphatidyl-choline 36:3
174	PC 36:4	Phosphatidyl-cholines	Phosphatidyl-choline 36:4
175	PC 36:5	Phosphatidyl-cholines	Phosphatidyl-choline 36:5
176	PC 36:6	Phosphatidyl-cholines	Phosphatidyl-choline 36:6
177	PC 38:0	Phosphatidyl-cholines	Phosphatidyl-choline 38:0
178	PC 38:1	Phosphatidyl-cholines	Phosphatidyl-choline 38:1
179	PC 38:3	Phosphatidyl-cholines	Phosphatidyl-choline 38:3
180	PC 38:4	Phosphatidyl-cholines	Phosphatidyl-choline 38:4
181	PC 38:5	Phosphatidyl-cholines	Phosphatidyl-choline 38:5
182	PC 38:6	Phosphatidyl-cholines	Phosphatidyl-choline 38:6
183	PC 40:2	Phosphatidyl-cholines	Phosphatidyl-choline 40:2
184	PC 40:3	Phosphatidyl-cholines	Phosphatidyl-choline 40:3
185	PC 40:4	Phosphatidyl-cholines	Phosphatidyl-choline 40:4
186	PC 40:5	Phosphatidyl-cholines	Phosphatidyl-choline 40:5
187	PC 40:6	Phosphatidyl-cholines	Phosphatidyl-choline 40:6
188	PC 42:2	Phosphatidyl-cholines	Phosphatidyl-choline 42:2
189	PC 42:5	Phosphatidyl-cholines	Phosphatidyl-choline 42:5
190	PC O-28:0	Phosphatidyl-cholines	Phosphatidyl-choline O-28:0
191	PC O-28:1	Phosphatidyl-cholines	Phosphatidyl-choline O-28:1
192	PC O-30:0	Phosphatidyl-cholines	Phosphatidyl-choline O-30:0
193	PC O-30:1	Phosphatidyl-cholines	Phosphatidyl-choline O-30:1
194	PC O-32:1	Phosphatidyl-cholines	Phosphatidyl-choline O-32:1
195	PC O-32:2	Phosphatidyl-cholines	Phosphatidyl-choline O-32:2
196	PC O-34:0	Phosphatidyl-cholines	Phosphatidyl-choline O-34:0
197	PC O-34:1	Phosphatidyl-cholines	Phosphatidyl-choline O-34:1
198	PC O-34:2	Phosphatidyl-cholines	Phosphatidyl-choline O-34:2

199	PC O-34:3	Phosphatidyl-cholines	Phosphatidyl-choline O-34:3
200	PC O-36:0	Phosphatidyl-cholines	Phosphatidyl-choline O-36:0
201	PC O-36:1	Phosphatidyl-cholines	Phosphatidyl-choline O-36:1
202	PC O-36:2	Phosphatidyl-cholines	Phosphatidyl-choline O-36:2
203	PC O-36:3	Phosphatidyl-cholines	Phosphatidyl-choline O-36:3
204	PC O-36:4	Phosphatidyl-cholines	Phosphatidyl-choline O-36:4
205	PC O-36:5	Phosphatidyl-cholines	Phosphatidyl-choline O-36:5
206	PC O-38:0	Phosphatidyl-cholines	Phosphatidyl-choline O-38:0
207	PC O-38:1	Phosphatidyl-cholines	Phosphatidyl-choline O-38:1
208	PC O-38:2	Phosphatidyl-cholines	Phosphatidyl-choline O-38:2
209	PC O-38:3	Phosphatidyl-cholines	Phosphatidyl-choline O-38:3
210	PC O-38:4	Phosphatidyl-cholines	Phosphatidyl-choline O-38:4
211	PC O-38:5	Phosphatidyl-cholines	Phosphatidyl-choline O-38:5
212	PC O-38:6	Phosphatidyl-cholines	Phosphatidyl-choline O-38:6
213	PC O-40:1	Phosphatidyl-cholines	Phosphatidyl-choline O-40:1
214	PC O-40:2	Phosphatidyl-cholines	Phosphatidyl-choline O-40:2
215	PC O-40:3	Phosphatidyl-cholines	Phosphatidyl-choline O-40:3
216	PC O-40:4	Phosphatidyl-cholines	Phosphatidyl-choline O-40:4
217	PC O-40:5	Phosphatidyl-cholines	Phosphatidyl-choline O-40:5
218	PC O-40:6	Phosphatidyl-cholines	Phosphatidyl-choline O-40:6
219	PC O-42:1	Phosphatidyl-cholines	Phosphatidyl-choline O-42:1
220	PC O-42:2	Phosphatidyl-cholines	Phosphatidyl-choline O-42:2
221	PC O-42:3	Phosphatidyl-cholines	Phosphatidyl-choline O-42:3
222	PE 28:0	Phosphatidyl-ethanolamines	Phosphatidyl-ethanolamine
223	PE 28:1	Phosphatidyl-ethanolamines	Phosphatidyl-ethanolamine
224	PE 30:0	Phosphatidyl-ethanolamines	Phosphatidyl-ethanolamine
225	PE 30:1	Phosphatidyl-ethanolamines	Phosphatidyl-ethanolamine
226	PE 31:0	Phosphatidyl-ethanolamines	Phosphatidyl-ethanolamine
227	PE 32:0	Phosphatidyl-ethanolamines	Phosphatidyl-ethanolamine
228	PE 32:1	Phosphatidyl-ethanolamines	Phosphatidyl-ethanolamine
229	PE 32:2	Phosphatidyl-ethanolamines	Phosphatidyl-ethanolamine
230	PE 33:0	Phosphatidyl-ethanolamines	Phosphatidyl-ethanolamine
231	PE 33:1	Phosphatidyl-ethanolamines	Phosphatidyl-ethanolamine
232	PE 33:2	Phosphatidyl-ethanolamines	Phosphatidyl-ethanolamine
233	PE 34:0	Phosphatidyl-ethanolamines	Phosphatidyl-ethanolamine
234	PE 34:1	Phosphatidyl-ethanolamines	Phosphatidyl-ethanolamine
235	PE 34:2	Phosphatidyl-ethanolamines	Phosphatidyl-ethanolamine
236	PE 34:3	Phosphatidyl-ethanolamines	Phosphatidyl-ethanolamine
237	PE 34:4	Phosphatidyl-ethanolamines	Phosphatidyl-ethanolamine
238	PE 35:1	Phosphatidyl-ethanolamines	Phosphatidyl-ethanolamine
239	PE 35:2	Phosphatidyl-ethanolamines	Phosphatidyl-ethanolamine
240	PE 35:3	Phosphatidyl-ethanolamines	Phosphatidyl-ethanolamine
241	PE 36:0	Phosphatidyl-ethanolamines	Phosphatidyl-ethanolamine
242	PE 36:1	Phosphatidyl-ethanolamines	Phosphatidyl-ethanolamine
243	PE 36:2	Phosphatidyl-ethanolamines	Phosphatidyl-ethanolamine
244	PE 36:3	Phosphatidyl-ethanolamines	Phosphatidyl-ethanolamine
245	PE 36:4	Phosphatidyl-ethanolamines	Phosphatidyl-ethanolamine
246	PE 36:5	Phosphatidyl-ethanolamines	Phosphatidyl-ethanolamine
247	PE 36:6	Phosphatidyl-ethanolamines	Phosphatidyl-ethanolamine
248	PE 38:0	Phosphatidyl-ethanolamines	Phosphatidyl-ethanolamine

249	PE 38:1	Phosphatidyl-ethanolamines	Phosphatidyl-ethanolamine
250	PE 38:2	Phosphatidyl-ethanolamines	Phosphatidyl-ethanolamine
251	PE 38:3	Phosphatidyl-ethanolamines	Phosphatidyl-ethanolamine
252	PE 38:4	Phosphatidyl-ethanolamines	Phosphatidyl-ethanolamine
253	PE 38:5	Phosphatidyl-ethanolamines	Phosphatidyl-ethanolamine
254	PE 38:6	Phosphatidyl-ethanolamines	Phosphatidyl-ethanolamine
255	PE 38:7	Phosphatidyl-ethanolamines	Phosphatidyl-ethanolamine
256	PE 40:1	Phosphatidyl-ethanolamines	Phosphatidyl-ethanolamine
257	PE 40:3	Phosphatidyl-ethanolamines	Phosphatidyl-ethanolamine
258	PE 40:4	Phosphatidyl-ethanolamines	Phosphatidyl-ethanolamine
259	PE 40:5	Phosphatidyl-ethanolamines	Phosphatidyl-ethanolamine
260	PE 40:6	Phosphatidyl-ethanolamines	Phosphatidyl-ethanolamine
261	PE 40:7	Phosphatidyl-ethanolamines	Phosphatidyl-ethanolamine
262	PE 40:8	Phosphatidyl-ethanolamines	Phosphatidyl-ethanolamine
263	PE 42:7	Phosphatidyl-ethanolamines	Phosphatidyl-ethanolamine
264	PE 42:8	Phosphatidyl-ethanolamines	Phosphatidyl-ethanolamine
265	PE 44:6	Phosphatidyl-ethanolamines	Phosphatidyl-ethanolamine
266	PE 44:7	Phosphatidyl-ethanolamines	Phosphatidyl-ethanolamine
267	PE 44:11	Phosphatidyl-ethanolamines	Phosphatidyl-ethanolamine
268	PE 44:12	Phosphatidyl-ethanolamines	Phosphatidyl-ethanolamine
269	PE P-16:0/14:0	Phosphatidyl-ethanolamines	Phosphatidyl-ethanolamine
270	PE P-16:0/15:0	Phosphatidyl-ethanolamines	Phosphatidyl-ethanolamine
271	PE P-16:0/16:0	Phosphatidyl-ethanolamines	Phosphatidyl-ethanolamine
272	PE P-16:0/16:1	Phosphatidyl-ethanolamines	Phosphatidyl-ethanolamine
273	PE P-16:0/18:1	Phosphatidyl-ethanolamines	Phosphatidyl-ethanolamine
274	PE P-16:0/18:2	Phosphatidyl-ethanolamines	Phosphatidyl-ethanolamine
275	PE P-16:0/18:3	Phosphatidyl-ethanolamines	Phosphatidyl-ethanolamine
276	PE P-16:0/20:3	Phosphatidyl-ethanolamines	Phosphatidyl-ethanolamine
277	PE P-16:0/20:4	Phosphatidyl-ethanolamines	Phosphatidyl-ethanolamine
278	PE P-16:0/20:5	Phosphatidyl-ethanolamines	Phosphatidyl-ethanolamine
279	PE P-16:0/22:4	Phosphatidyl-ethanolamines	Phosphatidyl-ethanolamine
280	PE P-16:0/22:5	Phosphatidyl-ethanolamines	Phosphatidyl-ethanolamine
281	PE P-16:0/22:6	Phosphatidyl-ethanolamines	Phosphatidyl-ethanolamine
282	PE P-18:0/14:0	Phosphatidyl-ethanolamines	Phosphatidyl-ethanolamine
283	PE P-18:0/16:0	Phosphatidyl-ethanolamines	Phosphatidyl-ethanolamine
284	PE P-18:0/16:1	Phosphatidyl-ethanolamines	Phosphatidyl-ethanolamine
285	PE P-18:0/17:1	Phosphatidyl-ethanolamines	Phosphatidyl-ethanolamine
286	PE P-18:0/18:1	Phosphatidyl-ethanolamines	Phosphatidyl-ethanolamine
287	PE P-18:0/18:2	Phosphatidyl-ethanolamines	Phosphatidyl-ethanolamine
288	PE P-18:0/18:3	Phosphatidyl-ethanolamines	Phosphatidyl-ethanolamine
289	PE P-18:0/20:1	Phosphatidyl-ethanolamines	Phosphatidyl-ethanolamine
290	PE P-18:0/20:2	Phosphatidyl-ethanolamines	Phosphatidyl-ethanolamine
291	PE P-18:0/20:3	Phosphatidyl-ethanolamines	Phosphatidyl-ethanolamine
292	PE P-18:0/20:4	Phosphatidyl-ethanolamines	Phosphatidyl-ethanolamine
293	PE P-18:0/20:5	Phosphatidyl-ethanolamines	Phosphatidyl-ethanolamine
294	PE P-18:0/22:1	Phosphatidyl-ethanolamines	Phosphatidyl-ethanolamine
295	PE P-18:0/22:2	Phosphatidyl-ethanolamines	Phosphatidyl-ethanolamine
296	PE P-18:0/22:3	Phosphatidyl-ethanolamines	Phosphatidyl-ethanolamine
297	PE P-18:0/22:4	Phosphatidyl-ethanolamines	Phosphatidyl-ethanolamine
298	PE P-18:0/22:5	Phosphatidyl-ethanolamines	Phosphatidyl-ethanolamine

299	PE P-18:0/22:6	Phosphatidyl-ethanolamines	Phosphatidyl-ethanolamine
300	PE P-18:1/18:1	Phosphatidyl-ethanolamines	Phosphatidyl-ethanolamine
301	PE P-18:1/18:2	Phosphatidyl-ethanolamines	Phosphatidyl-ethanolamine
302	PE P-18:1/20:4	Phosphatidyl-ethanolamines	Phosphatidyl-ethanolamine
303	PE P-18:1/20:5	Phosphatidyl-ethanolamines	Phosphatidyl-ethanolamine
304	PE P-18:1/22:6	Phosphatidyl-ethanolamines	Phosphatidyl-ethanolamine
305	PE P-20:0/14:0	Phosphatidyl-ethanolamines	Phosphatidyl-ethanolamine
306	PE P-20:0/16:0	Phosphatidyl-ethanolamines	Phosphatidyl-ethanolamine
307	PE P-20:0/16:1	Phosphatidyl-ethanolamines	Phosphatidyl-ethanolamine
308	PE P-20:0/18:1	Phosphatidyl-ethanolamines	Phosphatidyl-ethanolamine
309	PE P-20:0/18:2	Phosphatidyl-ethanolamines	Phosphatidyl-ethanolamine
310	PE P-20:0/20:4	Phosphatidyl-ethanolamines	Phosphatidyl-ethanolamine
311	PE P-20:0/20:5	Phosphatidyl-ethanolamines	Phosphatidyl-ethanolamine
312	PG 16:0_16:0	Phosphatidyl-glycerols	Phosphatidyl-glycerol
313	PG 16:0_16:1	Phosphatidyl-glycerols	Phosphatidyl-glycerol
314	PG 16:0_18:1	Phosphatidyl-glycerols	Phosphatidyl-glycerol
315	PG 16:0_18:2	Phosphatidyl-glycerols	Phosphatidyl-glycerol
316	PG 16:0_19:1	Phosphatidyl-glycerols	Phosphatidyl-glycerol
317	PG 16:0_20:3	Phosphatidyl-glycerols	Phosphatidyl-glycerol
318	PG 16:0_20:4	Phosphatidyl-glycerols	Phosphatidyl-glycerol
319	PG 16:0_20:5	Phosphatidyl-glycerols	Phosphatidyl-glycerol
320	PG 16:1_16:1	Phosphatidyl-glycerols	Phosphatidyl-glycerol
321	PG 16:1_18:1	Phosphatidyl-glycerols	Phosphatidyl-glycerol
322	PG 16:1_20:4	Phosphatidyl-glycerols	Phosphatidyl-glycerol
323	PG 16:2_18:1	Phosphatidyl-glycerols	Phosphatidyl-glycerol
324	PG 16:2_18:2	Phosphatidyl-glycerols	Phosphatidyl-glycerol
325	PG 16:3_18:1	Phosphatidyl-glycerols	Phosphatidyl-glycerol
326	PG 17:0_18:1	Phosphatidyl-glycerols	Phosphatidyl-glycerol
327	PG 17:1_18:1	Phosphatidyl-glycerols	Phosphatidyl-glycerol
328	PG 18:0_18:1	Phosphatidyl-glycerols	Phosphatidyl-glycerol
329	PG 18:1_18:1	Phosphatidyl-glycerols	Phosphatidyl-glycerol
330	PG 18:1_18:2	Phosphatidyl-glycerols	Phosphatidyl-glycerol
331	PG 18:1_20:0	Phosphatidyl-glycerols	Phosphatidyl-glycerol
332	PG 18:1_20:1	Phosphatidyl-glycerols	Phosphatidyl-glycerol
333	PG 18:1_20:2	Phosphatidyl-glycerols	Phosphatidyl-glycerol
334	PG 18:1_20:3	Phosphatidyl-glycerols	Phosphatidyl-glycerol
335	PG 18:1_20:4	Phosphatidyl-glycerols	Phosphatidyl-glycerol
336	PG 18:1_20:5	Phosphatidyl-glycerols	Phosphatidyl-glycerol
337	PG 18:1_22:0	Phosphatidyl-glycerols	Phosphatidyl-glycerol
338	PG 18:1_22:1	Phosphatidyl-glycerols	Phosphatidyl-glycerol
339	PG 18:1_22:2	Phosphatidyl-glycerols	Phosphatidyl-glycerol
340	PG 18:1_22:3	Phosphatidyl-glycerols	Phosphatidyl-glycerol
341	PG 18:1_22:4	Phosphatidyl-glycerols	Phosphatidyl-glycerol
342	PG 18:1_22:5	Phosphatidyl-glycerols	Phosphatidyl-glycerol
343	PI 14:0_18:1	Phosphatidyl-inositols	Phosphatidyl-inositol
344	PI 15:0_16:0	Phosphatidyl-inositols	Phosphatidyl-inositol
345	PI 15:1_16:0	Phosphatidyl-inositols	Phosphatidyl-inositol
346	PI 16:0_16:0	Phosphatidyl-inositols	Phosphatidyl-inositol
347	PI 16:0_17:2	Phosphatidyl-inositols	Phosphatidyl-inositol
348	PI 16:0_18:1	Phosphatidyl-inositols	Phosphatidyl-inositol

349	PI 16:0_20:4	Phosphatidyl-inositols	Phosphatidyl-inositol
350	PI 16:1_18:0	Phosphatidyl-inositols	Phosphatidyl-inositol
351	PI 16:1_18:1	Phosphatidyl-inositols	Phosphatidyl-inositol
352	PI 17:1_18:1	Phosphatidyl-inositols	Phosphatidyl-inositol
353	PI 18:0_18:0	Phosphatidyl-inositols	Phosphatidyl-inositol
354	PI 18:0_18:1	Phosphatidyl-inositols	Phosphatidyl-inositol
355	PI 18:0_18:2	Phosphatidyl-inositols	Phosphatidyl-inositol
356	PI 18:0_20:3	Phosphatidyl-inositols	Phosphatidyl-inositol
357	PI 18:0_20:4	Phosphatidyl-inositols	Phosphatidyl-inositol
358	PI 18:1_18:1	Phosphatidyl-inositols	Phosphatidyl-inositol
359	PI 18:1_18:2	Phosphatidyl-inositols	Phosphatidyl-inositol
360	PI 18:1_20:1	Phosphatidyl-inositols	Phosphatidyl-inositol
361	PI 18:1_20:2	Phosphatidyl-inositols	Phosphatidyl-inositol
362	PI 18:1_20:3	Phosphatidyl-inositols	Phosphatidyl-inositol
363	PI 18:1_20:4	Phosphatidyl-inositols	Phosphatidyl-inositol
364	PI 18:1_22:1	Phosphatidyl-inositols	Phosphatidyl-inositol
365	PI 18:1_22:6	Phosphatidyl-inositols	Phosphatidyl-inositol
366	PS 32:0	Phosphatidyl-serines	Phosphatidyl-serine 32:0
367	PS 34:1	Phosphatidyl-serines	Phosphatidyl-serine 34:1
368	PS 34:2	Phosphatidyl-serines	Phosphatidyl-serine 34:2
369	PS 36:1	Phosphatidyl-serines	Phosphatidyl-serine 36:1
370	PS 36:2	Phosphatidyl-serines	Phosphatidyl-serine 36:2
371	PS 36:3	Phosphatidyl-serines	Phosphatidyl-serine 36:3
372	PS 36:4	Phosphatidyl-serines	Phosphatidyl-serine 36:4
373	PS 36:5	Phosphatidyl-serines	Phosphatidyl-serine 36:5
374	PS 38:4	Phosphatidyl-serines	Phosphatidyl-serine 38:4
375	PS 38:5	Phosphatidyl-serines	Phosphatidyl-serine 38:5
376	PS 38:6	Phosphatidyl-serines	Phosphatidyl-serine 38:6
377	PS 38:7	Phosphatidyl-serines	Phosphatidyl-serine 38:7
378	PS 40:4	Phosphatidyl-serines	Phosphatidyl-serine 40:4
379	PS 40:5	Phosphatidyl-serines	Phosphatidyl-serine 40:5
380	PS 40:6	Phosphatidyl-serines	Phosphatidyl-serine 40:6
381	PS 40:7	Phosphatidyl-serines	Phosphatidyl-serine 40:7
382	PS 40:8	Phosphatidyl-serines	Phosphatidyl-serine 40:8
383	SPBP d14:1	Sphinganine and sphingosine	Sphingosine phosphate d14:1
384	SPBP d17:0	Sphinganine and sphingosine	Sphinganine phosphate d17:0
385	SPBP d18:1	Sphinganine and sphingosine	Sphingosine phosphate d18:1
386	SPB d14:0	Sphinganines and sphingosines	Sphinganine d14:0
387	SPB d18:0	Sphinganines and sphingosines	Sphinganine d18:0
388	SPB d18:1	Sphinganines and sphingosines	Sphingosine d18:1
389	SM 33:1	Sphingomyelins	Sphingomyelin 33:1
390	SM 34:1	Sphingomyelins	Sphingomyelin 34:1
391	SM 34:2	Sphingomyelins	Sphingomyelin 34:2
392	SM 35:1	Sphingomyelins	Sphingomyelin 35:1
393	SM 36:1	Sphingomyelins	Sphingomyelin 36:1
394	SM 36:2	Sphingomyelins	Sphingomyelin 36:2
395	SM 41:1	Sphingomyelins	Sphingomyelin 41:1
396	SM 41:2	Sphingomyelins	Sphingomyelin 41:2
397	SM 42:1	Sphingomyelins	Sphingomyelin 42:1
398	SM 42:2	Sphingomyelins	Sphingomyelin 42:2

399	TG 14:0_34:0	Triglycerides	Triacylglyceride 14:0_34:0
400	TG 14:0_34:1	Triglycerides	Triacylglyceride 14:0_34:1
401	TG 14:0_36:1	Triglycerides	Triacylglyceride 14:0_36:1
402	TG 14:0_36:2	Triglycerides	Triacylglyceride 14:0_36:2
403	TG 14:0_36:3	Triglycerides	Triacylglyceride 14:0_36:3
404	TG 16:0_32:0	Triglycerides	Triacylglyceride 16:0_32:0
405	TG 16:0_32:1	Triglycerides	Triacylglyceride 16:0_32:1
406	TG 16:0_33:1	Triglycerides	Triacylglyceride 16:0_33:1
407	TG 16:0_34:0	Triglycerides	Triacylglyceride 16:0_34:0
408	TG 16:0_34:1	Triglycerides	Triacylglyceride 16:0_34:1
409	TG 16:0_34:2	Triglycerides	Triacylglyceride 16:0_34:2
410	TG 16:0_35:1	Triglycerides	Triacylglyceride 16:0_35:1
411	TG 16:0_35:2	Triglycerides	Triacylglyceride 16:0_35:2
412	TG 16:0_35:3	Triglycerides	Triacylglyceride 16:0_35:3
413	TG 16:0_36:2	Triglycerides	Triacylglyceride 16:0_36:2
414	TG 16:1_28:0	Triglycerides	Triacylglyceride 16:1_28:0
415	TG 16:1_32:0	Triglycerides	Triacylglyceride 16:1_32:0
416	TG 16:1_32:1	Triglycerides	Triacylglyceride 16:1_32:1
417	TG 16:1_33:1	Triglycerides	Triacylglyceride 16:1_33:1
418	TG 16:1_34:0	Triglycerides	Triacylglyceride 16:1_34:0
419	TG 16:1_34:1	Triglycerides	Triacylglyceride 16:1_34:1
420	TG 16:1_34:2	Triglycerides	Triacylglyceride 16:1_34:2
421	TG 16:1_36:2	Triglycerides	Triacylglyceride 16:1_36:2
422	TG 17:0_34:2	Triglycerides	Triacylglyceride 17:0_34:2
423	TG 17:0_34:3	Triglycerides	Triacylglyceride 17:0_34:3
424	TG 17:1_34:1	Triglycerides	Triacylglyceride 17:1_34:1
425	TG 17:1_34:3	Triglycerides	Triacylglyceride 17:1_34:3
426	TG 18:0_30:0	Triglycerides	Triacylglyceride 18:0_30:0
427	TG 18:0_32:1	Triglycerides	Triacylglyceride 18:0_32:1
428	TG 18:1_30:0	Triglycerides	Triacylglyceride 18:1_30:0
429	TG 18:1_30:1	Triglycerides	Triacylglyceride 18:1_30:1
430	TG 18:1_32:0	Triglycerides	Triacylglyceride 18:1_32:0
431	TG 18:1_32:1	Triglycerides	Triacylglyceride 18:1_32:1
432	TG 18:1_32:2	Triglycerides	Triacylglyceride 18:1_32:2
433	TG 18:1_33:0	Triglycerides	Triacylglyceride 18:1_33:0
434	TG 18:1_33:1	Triglycerides	Triacylglyceride 18:1_33:1
435	TG 18:1_33:2	Triglycerides	Triacylglyceride 18:1_33:2
436	TG 18:1_33:3	Triglycerides	Triacylglyceride 18:1_33:3
437	TG 18:1_34:1	Triglycerides	Triacylglyceride 18:1_34:1
438	TG 18:1_34:2	Triglycerides	Triacylglyceride 18:1_34:2
439	TG 18:1_36:2	Triglycerides	Triacylglyceride 18:1_36:2
440	TG 18:2_33:2	Triglycerides	Triacylglyceride 18:2_33:2
441	TG 20:1_24:3	Triglycerides	Triacylglyceride 20:1_24:3
442	TG 20:1_31:0	Triglycerides	Triacylglyceride 20:1_31:0
443	Choline	Vitamins and cofactors	Choline

Table S2 Not Shown. The metabolic role (according to Biocrates Life Sciences) of the indicators shown in the corresponding line within Table 1.

1	Short-Chain Acyl-Coenzyme A Dehydrogenase (SCAD) Deficiency (NBS) is reported as Indicator of short-chain acyl-CoA dehydrogenase deficiency, a rare genetic condition that prevents the body from converting short-chain fatty acids into energy.
2	Lactate Dehydrogenase Activity is reported as an indicator of fermentation. Under hypoxic conditions, pyruvate is reduced by lactate dehydrogenase into lactate. In tumor cells, glucose is converted into pyruvate and further into lactate, even under sufficient oxygen conditions.
3	Isobutyryl-Coenzyme A Dehydrogenase Deficiency (NBS) is reported as an indicator of isobutyryl-CoA dehydrogenase deficiency, a condition that disrupts the breakdown of the amino acid valine.
5	Asparagine Synthesis is reported as Indicator of asparagine synthetase activity, an endogenous enzyme which catalyzes asparagine synthesis from aspartate. This enzyme has been described as a new potential biomarker of ovarian cancer and its downregulation has been shown to induce cell cycle arrest and inhibit cell proliferation of breast cancer.
6	Putrescine Synthesis is reported as indicator of ornithine decarboxylase activity, the rate-limiting enzyme in the polyamine synthesis decarboxylating ornithine to putrescine.
8	Polyamine Synthesis is reported as indicator of polyamine synthesis. First, ornithine is decarboxylated to putrescine by the rate-limiting enzyme ornithine decarboxylase, which is then further converted to spermidine and spermine by spermidine synthase and spermine synthase, respectively.
9	Sarcosine Synthesis from Choline is reported as indicator of sarcosine synthesis from choline. High levels of sarcosine have been shown to slightly reduce the risk of prostate cancer.
10	Glutaminolysis Rate is indicator of glutaminolysis rate, which is often markedly altered in cancer.
12	Betaine Synthesis is indicator of betaine synthesis from choline. Betaine acts as a methyl donor by carrying and donating methyl groups to facilitate chemical processes, e.g. for proper liver function, cellular replication, and detoxification reactions. Betaine insufficiency is associated with metabolic syndrome, lipid disorders and diabetes.
18	2-Methylbutyrylglycinuria (NBS) is Indicator of 2-methylbutyrylglycinuria, an inherited disorder that affects the catabolism of the amino acid isoleucine.
19	Taurine Synthesis is indicator of taurine synthesis from cysteine. Taurine is a highly abundant sulfonated amino acid in mammals and can be found in blood plasma and blood cells. It is essential for human infants and other species and plays a role in the development of central nervous, renal, cardiovascular, reproductive, and immune systems.
20	Gamma-Aminobutyric Acid Synthesis is indicator of glutamate decarboxylase activity converting the amino acid glutamate to gamma-aminobutyric acid.
21	Methionine Oxidation is fraction of sulfoxidized methionine relative to the unmodified methionine pool, which indicates systemic oxidative stress and biological ageing.
22	Glycine Synthesis is indicator of mitochondrial serine hydroxymethyltransferase (SHMT) activity converting serine to glycine. SHMT serves as a potential target for anti-malarial drugs and is a potential diagnostic marker for glioma and breast cancer.
23	Dihydrolipoamide Dehydrogenase Deficiency (NBS) is indicator of dihydrolipoamide dehydrogenase (DLD) deficiency, an inherited disease caused by the absence of the enzyme DLD, which is responsible for the degradation of the amino acids leucine, isoleucine and valine.
38	Ratio of Acetylcarnitine to Carnitine is indicator of beta-oxidation rate. It is a catabolic process in which fatty acids in the mitochondria of eukaryotes are degraded to acetyl-CoA, which then enters the citric acid cycle.
39	Sum of Solely Glucogenic Amino Acids is indicator for gluconeogenic activity and endogenous glucose production.
41	Sum of Non-Essential Amino Acids, indicates protein turn-over.

42	Cysteine Synthesis is an indicator of cysteine synthesis from serine or methionine. Cysteine is a non-essential sulfur-containing amino acid and important for protein synthesis, detoxification and diverse metabolic functions.
46	Carnosine Synthesis is an indicator of carnosine synthesis from histidine by carnosine synthetase activity.
47	Glutathione Constituents. Sum of constituents of the tripeptide glutathione (GSH), which is an antioxidant capable of preventing damage of cellular components caused by reactive oxygen species. GSH biosynthesis is facilitated in two steps: (i) glutamate cysteine ligase, the rate-limiting enzyme, fuses glutamate and cysteine, and (ii) glutathione synthetase adds glycine.
49	Sum of Conjugated Primary Bile Acids. Primary bile acids (BAs) are the BAs that are synthesized in the liver and then mostly conjugated to glycine or taurine to increase solubility and make them impermeable to cell membranes. Conjugated primary BAs are significantly elevated in patients with polycystic ovary syndrome. In addition, conjugated primary BAs were found to be elevated after antibiotic treatment.
51	51 Beta-Oxidation. Indicator of beta-oxidation rate. It is a catabolic process in which fatty acids in the mitochondria are degraded to acetyl-CoA, which then enters the citric acid cycle.
52	Sum of Taurine-Conjugated Bile Acids. After being synthesized from cholesterol, bile acids (BAs) are conjugated to glycine or taurine in the liver to increase solubility and make them impermeable to cell membranes. The content of taurine-conjugated BAs normally correlates with the content of glycine-conjugated BAs, unless the levels of taurine or glycine are abnormal.
53	Malonic Aciduria (NBS) is an indicator of malonic aciduria, a condition that prevents the body from converting certain fats into energy.
54	Sum of Sulfur-Containing Amino Acids. They contribute substantially to the maintenance and integrity of cell systems by influencing the redox state and the capacity to detoxify toxic compounds, free radicals and reactive oxygen species.
55	Short/Branched-Chain Acyl-Coenzyme A Dehydrogenase Deficiency (NBS) is an indicator of short/branched-chain acyl-CoA dehydrogenase deficiency, an autosomal recessive disorder of isoleucine catabolism.
79	Methylmalonic Acidemia (NBS) is an indicator of methylmalonic acidemia, an autosomal recessive metabolic disorder that disrupts the amino acid metabolism.

Table S3 Not Shown. Numbers generating graphic depicted in Figure 8

					Column A	Column B
Microscopic field	Mean NT	Mean Mico	Number nuclei NT	Number nuclei Mico	NT_ Mean normalized for nuclei number	Mico_ Mean normalized for nuclei number
**Field 1**	**18913**	**10542**	**6**	**5**	**3152.17**	**2108.40**
**Field 2**	**12224**	**10497**	**5**	**6**	**2444.80**	**1749.50**
**Field 3**	**14800**	**15289**	**6**	**3**	**2466.67**	**5096.33**
**Field 4**	**16583**	**13753**	**6**	**5**	**2763.83**	**2750.60**

**Field 5**	**20228**	**11007**	**5**	**7**	**4045.60**	**1572.43**
**Field 6**	**12214**	**14653**	**9**	**3**	**1357.11**	**4884.33**
**Field 7**	**19520**	**11566**	**6**	**5**	**3253.33**	**2313.20**
**Field 8**	**12796**	**13322**	**8**	**5**	**1599.50**	**2664.40**
**Field 9**	**13343**	**8928**	**4**	**3**	**3335.75**	**2976.00**
**Field 10**	**15567**	**10412**	**6**	**6**	**2594.50**	**1735.33**
**Field 11**	**15162**	**13633**	**3**	**14**	**5054.00**	**973.79**
**Field 12**	**14311**	**9894**	**9**	**8**	**1590.11**	**1236.75**
**Field 13**	**13655**	**12775**	**5**	**4**	**2731.00**	**3193.75**
**Field 14**	**11313**	**5891**	**4**	**6**	**2828.25**	**981.83**
**Field 15**	**7982**	**11129**	**5**	**4**	**1596.40**	**2782.25**
**Field 16**	**24435**	**4464**	**6**	**2**	**4072.50**	**2232.00**
**Field 17**	**36279**	**8526**	**12**	**7**	**3023.25**	**1218.00**
**Field 18**	**17417**	**13750**	**4**	**6**	**4354.25**	**2291.67**
**Field 19**	**21763**	**7622**	**5**	**6**	**4352.60**	**1270.33**
**Field 20**	**37892**	**6229**	**10**	**4**	**3789.20**	**1557.25**
**Field 21**	**18839**	**8788**	**4**	**2**	**4709.75**	**4394.00**
**Field 22**	**34468**	**6377**	**6**	**7**	**5744.67**	**911.00**
**Field 23**	**19627**	**5810**	**6**	**4**	**3271.17**	**1452.50**
**Field 24**	**9327**	**13023**	**4**	**4**	**2331.75**	**3255.75**
**Field 25**	**14830**	**9051**	**5**	**3**	**2966.00**	**3017.00**
**Field 26**	**21577**	**11643**	**2**	**2**	**10788.50**	**5821.50**
**Field 27**	**14047**	**17280**	**6**	**6**	**2341.17**	**2880.00**
**Field 28**	22969	12147	9	5	2552.11	2429.40
**Field 29**	18548	13750	7	5	2649.71	2750.00
**Field 30**	19147	4343	7	9	2735.29	482.56
				**Mean**	3349.83	2432.73
				**Dev. st**	3152.17	2108.40

				**t-Test column A vs. column B**	**0.0251**	
